# Supplementary material for: Perturbation-response genes reveal signaling footprints in cancer gene expression
Source: Nat Commun. 2018 Jan 2;9:20. doi: 10.1038/s41467-017-02391-6 (PMC5750219; doi:10.1038/s41467-017-02391-6)
Supplement: Supplementary file 1 — Supplementary Information [file 41467_2017_2391_MOESM1_ESM.pdf]

Supplementary Note

Supplementary Note 1: Database search terms for perturbations

Below, we list experimental evidence that our chosen pathway modulators indeed have the effect we assume in our model (PubMed IDs). We further provide our search term and all the ArrayExpress accessions we used to build our model.

- EGFR
  - Experimental evidence and secondary literature
    EGF[1], TGFα[1], Epiregulin[1], Heregulin[1], Neuregulin[1], Epigen[2], Beta-cellulin[1], Cetuximab[3], Amphiregulin[3], Gefinitib (Iressa)[3,4], Erlotinib (Tarceva)[3,4], Lapatinib[4], Tyrphostin AG1478[5], Trastuzumab (Herceptin)[3]
  - Search term
    (egf* OR "growth factor" OR TGFα OR epiregulin OR heregulin OR neuregulin OR epigen OR betacellulin OR amphiregulin OR iressa OR gefitinib OR cetuximab OR erlotinib OR lapatinib OR AG1478 OR trastuzumab OR herceptin) organism:sapiens samplecount:[4 TO 10000] raw:true exptype:"Transcription profiling"
  - Accessions
    E-GEOD-13168[6], E-GEOD-14256[7], E-GEOD-14987[8], E-GEOD-16179[9], E-GEOD-16659[10], E-GEOD-17948[11], E-GEOD-18232[12], E-GEOD-20854, E-GEOD-21618[13], E-GEOD-22288[14], E-GEOD-26732[15], E-GEOD-30516[16], E-GEOD-32217[17], E-GEOD-32975[18], E-GEOD-33442[19], E-GEOD-34104[20], E-GEOD-34652[21], E-GEOD-42781[22], E-GEOD-51212[23], E-GEOD-55823[24], E-GEOD-57156[25], E-GEOD-60880, E-GEOD-6462, E-MEXP-2065, E-MEXP-256, E-MEXP-440, E-MTAB-2091, E-TABM-440
- Hypoxia
  - Experimental evidence and secondary literature
    HIF-1[26]
  - Search term
    (hypoxia* OR HIF1 OR HIF-1) organism:sapiens samplecount:[4 TO 10000] raw:true exptype:"Transcription profiling"
  - Accessions
    E-GEOD-11904[27], E-GEOD-15583[28], E-GEOD-17353[29], E-GEOD-17714[30], E-GEOD-18494[31], E-GEOD-19197[32], E-GEOD-22282[33], E-GEOD-22469, E-GEOD-25452, E-GEOD-26820[34], E-GEOD-27523[35], E-GEOD-28603, E-GEOD-29406[36], E-GEOD-30979[37], E-GEOD-32100[38], E-GEOD-33115[39], E-GEOD-34112, E-GEOD-35819, E-GEOD-35973, E-GEOD-39042[40], E-GEOD-4086[41], E-GEOD-41999, E-GEOD-43608[42], E-GEOD-47009, E-GEOD-48002[43], E-GEOD-52315[44], E-GEOD-53012[45], E-GEOD-57613[46], E-GEOD-58049[47], E-GEOD-65168[48], E-GEOD-9649[49], E-TABM-948
- JAK-STAT
  - Experimental evidence and secondary literature
    Interferonβ/γ[50], IL-2[51], IL-3[51], IL-6[51,52], IL-10[51], IL-13[51], Prolaktin[50], EPO[51]
  - Search term
    (ifn* OR interferon OR JAK OR STAT OR prolaktin OR EPO OR IL-2 OR IL2 OR IL-3 OR IL3 OR IL-6 OR IL6 OR IL-10 OR IL10 OR IL-13 OR IL13) organism:sapiens samplecount:[4 TO 10000] raw:true exptype:"Transcription profiling"
  - Accessions
    E-GEOD-10685[53], E-GEOD-10943, E-GEOD-15743[54], E-GEOD-16452[55], E-GEOD-16755[56], E-GEOD-17477, E-GEOD-19182[57], E-GEOD-19392[58], E-GEOD-20272[59], E-GEOD-31193[60], E-GEOD-3183, E-GEOD-32387[61], E-GEOD-32407[62], E-GEOD-35601, E-GEOD-36287[63], E-GEOD-38147[64],

E-GEOD-43700[65], E-GEOD-440, E-GEOD-51402[66], E-GEOD-53213[67], E-GEOD-53751[68], E-GEOD-55510[69], E-GEOD-6092, E-GEOD-8515[70], E-GEOD-8687[71], E-GEOD-9481[72], E-MEXP-3746, E-MTAB-2091, E-MTAB-2477

- MAPK
  - Experimental evidence and secondary literature
    BRAF[73], RAF[73], MEK[73,74], ERK[73,74], PD-0325901[75], CI-1040[75], PLX4720[76], AZD6244[75], GSK1120212 (Trametinib)[75], U0126[74]
  - Search term
    (b-raf OR braf OR raf* OR mek* OR erk* OR PD*0325901 OR CI*1040 OR PLX* OR U0126 OR AZD6244 OR GSK1120212 OR *metinib OR PD*325901) organism:sapiens samplecount:[4 TO 10000] raw:true exptype:"Transcription profiling"
  - Accessions
    E-GEOD-10086[77], E-GEOD-12764[78,79], E-GEOD-13487[80], E-GEOD-13827, E-GEOD-14934[81], E-GEOD-15417[82], E-GEOD-17089[83], E-GEOD-18232[12], E-GEOD-20051[84], E-GEOD-24862[85], E-GEOD-29884, E-GEOD-31019[86], E-GEOD-32975[18], E-GEOD-35230[87], E-GEOD-41816[88], E-GEOD-42872[89], E-GEOD-45757[90], E-GEOD-45758[90], E-GEOD-50591[91], E-GEOD-50649[92], E-GEOD-51212[23], E-GEOD-53091, E-GEOD-58869
- NFkB
  - Experimental evidence and secondary literature
    TNFα pathway activation[93], Lipopolysaccharide (LPS)[94], Parthenidolide[95], Zymosan[96], BAY 11-7082[97]
  - Search term
    (LPS OR sa:lps OR sa:lipopolysaccharide OR pam3c* OR zymosan OR parthenolide OR sa:11-7082 OR sa:*salicyl*) organism:sapiens samplecount:[4 TO 10000] raw:true exptype:"Transcription profiling"
  - Accessions
    E-GEOD-10739[98], E-GEOD-14000[99], E-GEOD-14419, E-GEOD-16193[100], E-GEOD-16650, E-GEOD-19315[101], E-GEOD-19625[102], E-GEOD-20114[103], E-GEOD-22103[104], E-GEOD-23371[105], E-GEOD-24897[106], E-GEOD-25146[107], E-GEOD-25147[107], E-GEOD-25148[107], E-GEOD-26567, E-GEOD-29700[108], E-GEOD-32141[109], E-GEOD-40885, E-GEOD-42358[110], E-GEOD-42660[111], E-GEOD-43596[112], E-GEOD-46236[113], E-GEOD-46914[114], E-GEOD-59472[115], E-GEOD-7538[116], E-GEOD-9916, E-MEXP-1103, E-MEXP-2577, E-MEXP-3931, E-MTAB-1393, E-TABM-868
- p53
  - Experimental evidence and secondary literature
    DNA double strand breaks[117], Ionizing radiation[118], Nutlin-3a (MDM2 inhibition)[119,120], RITA (MDM2 inhibition)[121], Doxorubicin[120], Cisplatin[122,123], Carboplatin[123], Oxaliplatin[123]
  - Search term
    (sa:*p53 OR sa:mdm2 OR sa:nutlin* OR sa:*radiation OR sa:*radiated OR sa:"dna damage" OR sa:ddr OR sa:*rubicin OR sa:*platin OR sa:docetaxel OR sa:gamma) organism:sapiens samplecount:[4 TO 10000] raw:true exptype:"Transcription profiling"
  - Accessions
    E-GEOD-11578[124], E-GEOD-15658, E-GEOD-18552[125], E-GEOD-30137[126], E-GEOD-30174[127], E-GEOD-30753, E-GEOD-30988[128], E-GEOD-35006[129], E-GEOD-46642[130], E-GEOD-56640[131], E-MEXP-2623, E-MEXP-390, E-MTAB-1403
- PI3K
  - Experimental evidence and secondary literature
    PIK3CA H1047R[132], Ras G12V/Y40C[81], PTEN[133], BKM120[134], LY294002[135,136], GSK1059615[134], PI-103[136], GDC941[134], BEZ235[134],

- Search term
  (SF1126 OR LY294002 OR TGR*1202 OR SAR2454* OR GSK1059615 OR 80*6946 OR Perifosine OR Idelalisib OR PI3K OR PIK3* OR PTEN OR BEZ235 OR RP6530 OR GDC*941 OR INK1117) organism:sapiens samplecount:[4 TO 10000] raw:true exptype:"Transcription profiling"
- Accessions
  E-GEOD-14934[81], E-GEOD-17785, E-GEOD-32975[18], E-GEOD-33643[137], E-GEOD-38255, E-GEOD-40564[138], E-GEOD-42762[139], E-GEOD-45276[140], E-GEOD-51212[23], E-GEOD-53309[141], E-GEOD-6263[142], E-GEOD-9601[143], E-MEXP-3640

- TGFb
  - Experimental evidence and secondary literature
    TGFβ ligand[144,145], BMP ligands (BMP2, BMP4, BMP6)[146],
  - Search term
    (tgf OR "tgf-*" OR "bmp*" OR "SMAD*" "transforming growth factor" OR "A 83-01" OR "A-83-01" OR "A83-01" OR "D 4476" OR "D4476" OR "D-4476" OR "GW 788388" OR "GW788388" OR "GW-788388" OR "LY 364947" OR "LY-364947" OR "LY364947" OR "R 268712" OR "R-268712" OR "R268712" OR RepSox OR "SB 431542" OR "SB-431542" OR "SB431542" OR "SB 505124" OR "SB-505124" OR "SB505124" OR "SB 525334" OR "SB-525334" OR "SB525334" OR "SD 208" OR "SD208" OR "SD-208") organism:sapiens samplecount:[4 TO 10000] raw:true exptype:"Transcription profiling"
  - Accessions
    E-GEOD-10311[147], E-GEOD-16111, E-GEOD-20671[148], E-GEOD-26241[149], E-GEOD-26351[150], E-GEOD-27526[151], E-GEOD-28074[152], E-GEOD-40266[153], E-GEOD-46019[154], E-GEOD-5457, E-GEOD-54756, E-GEOD-60135[155], E-GEOD-63383[156], E-GEOD-7144, E-MEXP-2645

- TNFa
  - Experimental evidence and secondary literature
    TNFα[157,158]
  - Search term
    (tnf* OR "necrosis factor") organism:sapiens samplecount:[4 TO 10000] raw:true exptype:"Transcription profiling"
  - Accessions
    E-GEOD-11115, E-GEOD-11467[159], E-GEOD-12161[160], E-GEOD-12548[161], E-GEOD-13837[162], E-GEOD-16650, E-GEOD-24422[163], E-GEOD-2638[164], E-GEOD-2639, E-GEOD-26868[165], E-GEOD-28548, E-GEOD-32975[18], E-GEOD-36287[63], E-GEOD-37139[166], E-GEOD-38351[167], E-GEOD-40413[168], E-GEOD-43685[169], E-GEOD-43738[170], E-GEOD-59237[171], E-GEOD-59472[115], E-GEOD-65707[172], E-GEOD-8166[173], E-MEXP-3540, E-MTAB-1027, E-MTAB-1312, E-MTAB-2091, E-SGRP-3

- Trail
  - Experimental evidence and secondary literature
    Recombinant Trail[174], FasL[174], Caspase 8[174], ABT-737[175], ABT-199[176], GX15-070 (Obatoclax)[175]
  - Search term
    (rtrail OR trail* OR fas OR fasl) organism:sapiens samplecount:[4 TO 10000] raw:true exptype:"Transcription profiling" array:(A-AFFY-33 OR A-AFFY-44 OR A-AFFY-37 OR A-AFFY-141) OR (ABT-737 OR ABT-199 OR obatoclax OR GX15-070 OR casp8 OR caspase) organism:sapiens samplecount:[4 TO 10000] raw:true exptype:"Transcription profiling"
  - Accessions
    E-GEOD-29955[177], E-GEOD-36149[178], E-GEOD-36572, E-GEOD-62533[179], E-GEOD-8346

- VEGF

– Experimental evidence and secondary literature
VEGF[180], PGF/PDGF[181], FGF[182,183], Sunitinib (Sutent)[184], Pazopanib (Votrient)[184], Everolimus (Afinitor)[185], Bevacizumab (Avastin)[184], Sorafenib (Nexavar)[184], Withaferin[186],

– Search term
(VEGF* OR sunitinib OR nexavar OR sutent OR pazopanib OR OR afinitor OR votrient OR Bevacizumab OR sorafenib OR PDGF* OR PGF OR FGF OR Withaferin) organism:sapiens samplecount:[4 TO 10000] raw:true exptype:"Transcription profiling"

– Accessions
E-GEOD-14256[7], E-GEOD-18913[187], E-GEOD-19098[188], E-GEOD-1923[189], E-GEOD-29368[190], E-GEOD-42001, E-GEOD-48841[191], E-GEOD-52488[192], E-GEOD-53633[193], E-GEOD-58203[194], E-GEOD-7403[195], E-MTAB-2091

Supplementary Tables

Supplementary Table 1: Area under the ROC curve for perturbation experiments. Pathway scores derived by different pathway methods, known pathway perturbations are True Positives.

inferred	PROGENy	Z-score	SPEED (our data)	SPEED (web)	EPSA (our data)	GSEA (our genes)
EGFR	0.81	0.50	0.71	0.59	0.78	0.37
Hypoxia	0.96	0.51	0.95	NA	0.92	0.94
JAK-STAT	0.87	0.51	0.95	0.76	0.80	0.90
MAPK	0.81	0.50	0.17	0.13	0.38	0.88
NFkB	0.82	0.50	0.94	NA	0.87	0.87
p53	0.93	0.52	0.82	NA	0.87	0.92
PI3K	0.79	0.52	0.20	0.72	0.22	0.14
TGFb	0.92	0.49	0.85	0.48	0.85	0.88
TNFa	0.93	0.50	0.98	0.78	0.88	0.98
Trail	0.60	0.52	0.98	NA	0.88	0.71
VEGF	0.78	0.49	0.83	0.36	0.70	0.25

Supplementary Table 2: Pathway mapping for different methods

(a) Gene Ontology. GO categories (ID and name) shown for the 11 pathways we investigate.

2*Pathway	Gene Ontology	
	ID	Name
EGFR	GO:0007259	ERBB signaling pathway
Hypoxia	GO:0071456	cellular response to hypoxia
JAK-STAT	GO:0007259	JAK-STAT signal transduction
MAPK	GO:0000165	MAPK cascade
NFkB	GO:0038061	NIK/NF-kappaB signaling
p53	GO:0030330	DNA damage response, signal transduction by p53 class mediator
PI3K	GO:0014065	phosphatidylinositol 3-kinase signaling
TNFa	GO:0033209	tumor necrosis factor-mediated signaling pathway
TGFb	GO:0007179	transforming growth factor beta receptor signaling pathway
Trail	GO:0036462	TRAIL-activated apoptotic signaling pathway
VEGF	GO:0038084	vascular endothelial growth factor signaling pathway

(b) Reactome and Pathifier. Listed are reactome pathway names that were used either for GSVA pathway scores or Pathifier scores using the respective R packages. If more than one pathway is listed all of them were used in the gene set.

Pathway	Name
2*EGFR	Signaling by EGFR
	Signaling by EGFR in Cancer
Hypoxia	Cellular response to hypoxia
3*JAK-STAT	Signaling by Interleukins
	Interferon Signaling
	Signalling to STAT3
MAPK	Signalling to ERKs
2*NFkB	TAK1 activates NFkB:w: by phosphorylation and activation of IKKs complex
	RIP-mediated NFkB activation via ZBP1
p53	Transcriptional Regulation by TP53
4*PI3K	PI3K Cascade
	Constitutive Signaling by Aberrant PI3K in Cancer
	PI3K/AKT Signaling in Cancer
	PI3K/AKT activation
TNFa	TNF signaling
TGFb	Signaling by TGF-beta Receptor Complex
Trail	TRAIL signaling
VEGF	Signaling by VEGF

(c) Signaling Pathway Impact Analysis. KEGG pathway IDs and names that SPIA relies on are listed (this is part of the SPIA R package).

2*Pathway	SPIA	
	ID	Name
EGFR	04012	ErbB signaling pathway
Hypoxia	-	-
JAK-STAT	04630	Jak-STAT signaling pathway
MAPK	04010	MAPK signaling pathway
NFkB	04064	NF-kappa B signaling pathway
p53	-	-
PI3K	04150	mTOR signaling pathway
TNFa	-	-
TGFb	04350	TGF-beta signaling pathway
Trail	04210	Apoptosis
VEGF	04370	VEGF signaling pathway

(d) PARADIGM. This method is different as it performs inference on a signaling network instead of providing pathways itself. We used the network the TCGA is using in their analyses[1]. This network has activity nodes for pathways and important processes that we used here, as listed.

Pathway	PARADIGM
EGFR	epidermal growth factor receptor activity (abstract)
Hypoxia	response to hypoxia (abstract)
JAK-STAT	STAT-1-3-5-active
MAPK	MEK-1-2-active
NFkB	NFkB Complex (complex)
p53	response to DNA damage stimulus (abstract)
PI3K	PIK3CA
TNFa	tumor necrosis factor receptor activity (abstract)
TGFb	SMAD1-5-8-active
Trail	induction of apoptosis (abstract)
VEGF	platelet-derived growth factor receptor activity (abstract)

(e) Signatures published and validated in Gatza et al. (2009)

Pathway	Gatza (2009)
EGFR	EGFR
Hypoxia	HYPOXIA
JAK-STAT	STAT1, STAT3, IFNA, IFNG
MAPK	PROLIFERATION, PROLIFERATION (PAM50)
p53	P53, P53 MUT/P53 WT CORR
PI3K	PI3K, PIK3CA
TNFa	TNFA
TGFb	TGFB
VEGF	VEGF/HYPOXIA

Supplementary Table 3: FDR-adjusted p-values for mutation/copy number associations with pathway scores obtained by PROGENy. Associations using TCGA data corrected for cancer type. Mutated genes included in table if any method has FDR $< 10^{-7}$, copy number alterations for related genes (e.g. ERBB2_amp for EGFR).

Method	Subset	EGFR	Hypoxia	JAK.STAT	MAPK	NFkB	p53	PI3K	TGFb	TNFa	Trail	VEGF
Gene Ontology	ARID1A	n.s.	n.s.	n.s.	n.s.	n.s.	1.6e-07	n.s.	n.s.	n.s.	n.s.	n.s.
Pathifier	ARID1A	n.s.	n.s.	2.7e-09	n.s.	n.s.	n.s.	n.s.	n.s.	n.s.	n.s.	n.s.
Iorio (2016)	ARID1A	n.s.	n.s.	n.s.	n.s.	n.s.	n.s.	3.0e-04	n.s.	n.s.	n.s.	n.s.
PROGENy	ASH1L	n.s.	7.3e-04	n.s.	n.s.	n.s.	n.s.	n.s.	n.s.	n.s.	n.s.	n.s.
Reactome	ASH1L	n.s.	n.s.	n.s.	n.s.	n.s.	n.s.	n.s.	4.4e-04	n.s.	n.s.	n.s.
Iorio (2016)	ASH1L	9.6e-08	n.s.	n.s.	4.4e-06	n.s.	n.s.	1.6e-05	n.s.	n.s.	n.s.	n.s.
Gatza (2009)	ASH1L	n.s.	1.5e-04	n.s.	n.s.	n.s.	n.s.	5.2e-08	n.s.	n.s.	n.s.	5.1e-04
PROGENy	BRAF	1.3e-11	n.s.	7.3e-04	1.1e-18	4.8e-16	n.s.	n.s.	n.s.	2.1e-17	n.s.	n.s.
Gene Ontology	BRAF	3.8e-04	n.s.	1.6e-13	3.3e-07	n.s.	3.2e-08	1.9e-08	n.s.	1.2e-04	1.3e-10	n.s.
Reactome	BRAF	n.s.	n.s.	6.2e-18	1.3e-09	1.4e-13	n.s.	n.s.	n.s.	n.s.	1.8e-10	4.5e-04
SPIA	BRAF	n.s.	n.s.	3.0e-04	3.0e-04	1.5e-05	n.s.	n.s.	n.s.	n.s.	2.6e-04	n.s.
Pathifier	BRAF	1.3e-07	5.1e-04	n.s.	n.s.	1.7e-07	n.s.	1.7e-07	n.s.	8.3e-04	1.0e-08	1.6e-15
PARADIGM	BRAF	n.s.	n.s.	n.s.	3.0e-06	n.s.	n.s.	n.s.	n.s.	n.s.	n.s.	n.s.
Iorio (2016)	BRAF	9.7e-05	n.s.	n.s.	n.s.	n.s.	n.s.	n.s.	n.s.	6.1e-09	n.s.	n.s.
Gatza (2009)	BRAF	2.5e-12	n.s.	8.6e-20	3.0e-04	n.s.	n.s.	1.4e-07	n.s.	3.0e-18	n.s.	n.s.
Gatza (2009)	BRAF_amp	n.s.	n.s.	n.s.	1.8e-04	n.s.	n.s.	n.s.	n.s.	n.s.	n.s.	n.s.
PROGENy	CDH1	n.s.	n.s.	n.s.	n.s.	n.s.	n.s.	1.3e-09	n.s.	n.s.	n.s.	n.s.
Gene Ontology	CDH1	n.s.	n.s.	n.s.	n.s.	1.0e-08	n.s.	2.3e-04	n.s.	n.s.	n.s.	n.s.
Reactome	CDH1	n.s.	n.s.	n.s.	n.s.	n.s.	3.3e-07	n.s.	1.0e-07	n.s.	n.s.	n.s.
SPIA	CDH1	n.s.	n.s.	9.4e-06	n.s.	n.s.	n.s.	n.s.	n.s.	n.s.	n.s.	n.s.
Pathifier	CDH1	n.s.	4.5e-04	n.s.	n.s.	n.s.	3.8e-07	n.s.	n.s.	n.s.	n.s.	n.s.
Iorio (2016)	CDH1	n.s.	n.s.	n.s.	1.7e-06	n.s.	n.s.	1.6e-06	n.s.	n.s.	n.s.	6.4e-08
Gatza (2009)	CDH1	n.s.	n.s.	n.s.	6.9e-13	n.s.	n.s.	2.0e-11	n.s.	n.s.	n.s.	1.9e-05
SPIA	CTNNB1	n.s.	n.s.	n.s.	1.5e-05	n.s.	n.s.	n.s.	3.8e-06	n.s.	n.s.	n.s.
Pathifier	CTNNB1	3.9e-13	n.s.	n.s.	n.s.	n.s.	n.s.	2.4e-28	n.s.	n.s.	n.s.	n.s.
PROGENy	EGFR_amp	2.6e-10	2.3e-04	n.s.	4.4e-20	2.3e-04	n.s.	n.s.	n.s.	9.4e-05	n.s.	3.6e-06
Gene Ontology	EGFR_amp	n.s.	n.s.	n.s.	n.s.	n.s.	n.s.	n.s.	n.s.	n.s.	9.4e-05	n.s.
Reactome	EGFR_amp	n.s.	n.s.	n.s.	n.s.	n.s.	n.s.	n.s.	n.s.	8.9e-05	n.s.	n.s.
SPIA	EGFR_amp	4.7e-12	n.s.	n.s.	n.s.	n.s.	n.s.	n.s.	n.s.	n.s.	n.s.	n.s.
Gatza (2009)	EGFR_amp	n.s.	n.s.	n.s.	1.7e-06	n.s.	4.2e-06	3.9e-04	n.s.	n.s.	n.s.	8.2e-09
PROGENy	ERBB2_amp	n.s.	n.s.	n.s.	n.s.	n.s.	n.s.	n.s.	n.s.	n.s.	6.0e-05	n.s.
Gene Ontology	ERBB2_amp	n.s.	n.s.	2.5e-05	n.s.	1.8e-04	n.s.	n.s.	n.s.	n.s.	2.9e-05	n.s.
Reactome	ERBB2_amp	n.s.	n.s.	n.s.	n.s.	n.s.	6.1e-04	n.s.	n.s.	n.s.	n.s.	n.s.
SPIA	ERBB2_amp	n.s.	n.s.	n.s.	1.1e-10	5.1e-11	n.s.	n.s.	n.s.	n.s.	n.s.	n.s.
Iorio (2016)	ERBB2_amp	n.s.	n.s.	1.4e-05	2.6e-13	n.s.	n.s.	8.1e-06	2.0e-05	n.s.	n.s.	n.s.
Gatza (2009)	ERBB2_amp	n.s.	n.s.	n.s.	5.3e-16	n.s.	1.4e-07	1.1e-06	n.s.	n.s.	n.s.	n.s.
Reactome	GATA3	n.s.	n.s.	n.s.	n.s.	n.s.	4.4e-04	n.s.	n.s.	n.s.	n.s.	n.s.
SPIA	GATA3	n.s.	n.s.	n.s.	1.3e-06	n.s.	n.s.	n.s.	n.s.	n.s.	n.s.	n.s.
Iorio (2016)	GATA3	n.s.	n.s.	n.s.	n.s.	n.s.	n.s.	4.7e-05	n.s.	n.s.	n.s.	n.s.
Gatza (2009)	GATA3	n.s.	n.s.	n.s.	n.s.	n.s.	3.3e-13	n.s.	n.s.	n.s.	n.s.	n.s.
Reactome	KEAP1	n.s.	n.s.	n.s.	n.s.	n.s.	3.2e-05	n.s.	n.s.	n.s.	n.s.	n.s.
Pathifier	KEAP1	n.s.	n.s.	n.s.	n.s.	n.s.	6.1e-19	n.s.	n.s.	n.s.	n.s.	n.s.
Iorio (2016)	KEAP1	n.s.	n.s.	n.s.	n.s.	n.s.	n.s.	1.7e-06	n.s.	n.s.	n.s.	n.s.
PROGENy	KRAS	1.3e-09	n.s.	n.s.	5.4e-11	n.s.	n.s.	n.s.	n.s.	n.s.	n.s.	n.s.
Reactome	KRAS	n.s.	5.5e-04	n.s.	n.s.	n.s.	n.s.	n.s.	1.3e-04	n.s.	n.s.	n.s.
Gatza (2009)	KRAS	4.6e-04	5.3e-04	n.s.	n.s.	n.s.	n.s.	n.s.	n.s.	n.s.	n.s.	n.s.
PROGENy	KRAS_amp	2.4e-06	n.s.	n.s.	1.3e-08	n.s.	n.s.	n.s.	n.s.	n.s.	n.s.	n.s.
Reactome	KRAS_amp	n.s.	n.s.	n.s.	n.s.	n.s.	n.s.	n.s.	4.3e-04	n.s.	n.s.	n.s.
SPIA	KRAS_amp	n.s.	n.s.	n.s.	n.s.	n.s.	n.s.	n.s.	n.s.	n.s.	n.s.	1.3e-07
Pathifier	KRAS_amp	n.s.	n.s.	n.s.	7.5e-05	n.s.	n.s.	n.s.	n.s.	n.s.	n.s.	n.s.
Gatza (2009)	KRAS_amp	n.s.	n.s.	n.s.	3.1e-06	n.s.	8.7e-05	7.5e-04	n.s.	n.s.	n.s.	n.s.
PROGENy	NFE2L2	n.s.	n.s.	n.s.	n.s.	n.s.	1.1e-09	n.s.	n.s.	n.s.	n.s.	n.s.
Reactome	NFE2L2	n.s.	n.s.	n.s.	n.s.	n.s.	4.8e-11	n.s.	n.s.	n.s.	n.s.	n.s.
PROGENy	NRAS	2.2e-04	n.s.	n.s.	n.s.	2.8e-05	n.s.	n.s.	n.s.	3.6e-07	n.s.	n.s.
Gene Ontology	NRAS	n.s.	n.s.	1.2e-08	2.8e-05	n.s.	n.s.	9.4e-05	n.s.	n.s.	7.7e-06	n.s.
Reactome	NRAS	n.s.	n.s.	8.4e-10	n.s.	5.5e-05	n.s.	7.2e-04	n.s.	n.s.	2.1e-04	n.s.
SPIA	NRAS	n.s.	n.s.	6.2e-04	n.s.	n.s.	n.s.	n.s.	n.s.	n.s.	n.s.	n.s.
Pathifier	NRAS	n.s.	n.s.	n.s.	n.s.	n.s.	n.s.	n.s.	n.s.	n.s.	n.s.	1.3e-05
Gatza (2009)	NRAS	2.4e-06	n.s.	1.7e-11	n.s.	n.s.	n.s.	n.s.	n.s.	1.4e-08	n.s.	n.s.
PROGENy	PIK3CA	n.s.	n.s.	n.s.	4.3e-05	n.s.	1.8e-07	n.s.	n.s.	n.s.	n.s.	n.s.
Gene Ontology	PIK3CA	n.s.	n.s.	n.s.	n.s.	n.s.	n.s.	6.0e-04	n.s.	n.s.	n.s.	n.s.
Gatza (2009)	PIK3CA	n.s.	n.s.	n.s.	1.5e-04	n.s.	3.7e-10	n.s.	n.s.	n.s.	n.s.	3.3e-06
PROGENy	PIK3CA_amp	6.9e-04	2.3e-07	n.s.	n.s.	n.s.	n.s.	n.s.	n.s.	n.s.	n.s.	n.s.
Gene Ontology	PIK3CA_amp	n.s.	n.s.	n.s.	n.s.	n.s.	n.s.	n.s.	n.s.	n.s.	n.s.	7.5e-07
SPIA	PIK3CA_amp	n.s.	n.s.	n.s.	2.7e-06	3.3e-05	n.s.	n.s.	n.s.	n.s.	n.s.	n.s.
PARADIGM	PIK3CA_amp	n.s.	n.s.	n.s.	n.s.	n.s.	n.s.	1.2e-142	n.s.	n.s.	n.s.	n.s.
Iorio (2016)	PIK3CA_amp	n.s.	n.s.	2.3e-07	8.7e-12	n.s.	n.s.	n.s.	n.s.	n.s.	n.s.	n.s.
Gatza (2009)	PIK3CA_amp	n.s.	n.s.	n.s.	2.2e-28	n.s.	2.3e-21	3.1e-20	n.s.	n.s.	n.s.	7.0e-07
PROGENy	PTEN_del	n.s.	3.4e-07	n.s.	n.s.	n.s.	n.s.	n.s.	n.s.	n.s.	n.s.	n.s.
Gene Ontology	PTEN_del	n.s.	n.s.	n.s.	n.s.	n.s.	1.6e-05	4.7e-05	n.s.	2.8e-05	n.s.	n.s.
Reactome	PTEN_del	1.4e-06	n.s.	n.s.	n.s.	n.s.	n.s.	6.6e-15	n.s.	n.s.	n.s.	n.s.
Iorio (2016)	PTEN_del	n.s.	n.s.	n.s.	8.5e-07	n.s.	n.s.	n.s.	n.s.	n.s.	n.s.	n.s.
Gatza (2009)	PTEN_del	n.s.	n.s.	n.s.	7.9e-16	n.s.	n.s.	8.4e-15	2.2e-04	n.s.	n.s.	2.0e-09
Gatza (2009)	RB1	n.s.	n.s.	n.s.	5.4e-10	n.s.	n.s.	2.8e-11	n.s.	n.s.	n.s.	n.s.
PROGENy	TP53	n.s.	2.1e-17	2.7e-05	1.5e-07	3.9e-04	5.2e-65	2.4e-21	n.s.	3.7e-04	n.s.	n.s.
Gene Ontology	TP53	n.s.	7.7e-04	n.s.	6.6e-05	6.2e-13	n.s.	1.1e-11	n.s.	4.6e-07	n.s.	n.s.
Reactome	TP53	9.3e-04	n.s.	n.s.	n.s.	n.s.	n.s.	1.4e-13	2.5e-08	n.s.	n.s.	n.s.
Pathifier	TP53	8.5e-18	9.4e-13	n.s.	7.5e-05	1.7e-13	n.s.	3.7e-07	n.s.	n.s.	n.s.	3.7e-04
PARADIGM	TP53	n.s.	n.s.	n.s.	n.s.	n.s.	n.s.	6.6e-08	n.s.	n.s.	n.s.	n.s.
Iorio (2016)	TP53	n.s.	n.s.	n.s.	2.9e-16	n.s.	n.s.	n.s.	n.s.	n.s.	n.s.	6.1e-09
Gatza (2009)	TP53	n.s.	n.s.	n.s.	5.6e-58	n.s.	4.3e-105	1.1e-32	n.s.	n.s.	n.s.	1.3e-56
PROGENy	TP53_del	n.s.	n.s.	n.s.	n.s.	n.s.	3.2e-05	n.s.	n.s.	n.s.	n.s.	n.s.
Iorio (2016)	TP53_del	n.s.	n.s.	n.s.	1.9e-04	n.s.	n.s.	n.s.	n.s.	n.s.	n.s.	n.s.
Gatza (2009)	TP53_del	n.s.	n.s.	n.s.	n.s.	n.s.	9.8e-07	n.s.	n.s.	n.s.	n.s.	n.s.

Supplementary Table 4: Stratification statistics: subsets and numbers for figure 4c. Shown is the combination between a mutation/pathway, the number of cell lines, and median drug response in each subset. Subsets are defined by: (1) Whether a mutation is present (mut) or not (wt), or disregarding mutational status (blank). (2) Whether the pathway score is in the top (active) or bottom quartile (inactive), or neither (blank) of the subset defined by mutations.

Treatment	Mutation	Pathway	Number of cell lines	Median DR [log uM]
BRAF + Dabrafenib			756	3.78
BRAF + Dabrafenib		active	189	3.03
BRAF + Dabrafenib		average	378	3.86
BRAF + Dabrafenib		inactive	189	4.18
BRAF + Dabrafenib	mut		75	-1.57
BRAF + Dabrafenib	mut	active	19	-2.55
BRAF + Dabrafenib	mut	inactive	19	2.18
BRAF + Dabrafenib	wt		681	3.93
BRAF + Dabrafenib	wt	active	170	3.70
BRAF + Dabrafenib	wt	inactive	170	4.25
MAPK + AZ628			756	1.97
MAPK + AZ628		active	189	-0.59
MAPK + AZ628		average	378	1.75
MAPK + AZ628		inactive	189	2.75
MAPK + AZ628	mut		222	0.41
MAPK + AZ628	mut	active	56	-0.83
MAPK + AZ628	mut	inactive	56	0.99
MAPK + AZ628	wt		534	2.42
MAPK + AZ628	wt	active	134	1.63
MAPK + AZ628	wt	inactive	134	2.93
MAPK + Trametinib			756	-0.97
MAPK + Trametinib		active	189	-3.11
MAPK + Trametinib		average	378	-0.78
MAPK + Trametinib		inactive	189	1.14
MAPK + Trametinib	mut		222	-3.26
MAPK + Trametinib	mut	active	56	-4.38
MAPK + Trametinib	mut	inactive	56	-0.42
MAPK + Trametinib	wt		534	-0.28
MAPK + Trametinib	wt	active	134	-1.59
MAPK + Trametinib	wt	inactive	134	1.61

Supplementary Table 5: Stratification statistics: significance tests for figure 4c. For the same subsets, results of the Mann-Whitney U test between different quartiles of the pathway score within different subsets defined by mutational status with p-value as indicated. Difference in mutations indicated by wt (wild-type), mut (mutated), or blank (any). Inferred pathway activity is indicated by + (top quartile) − (bottom quartile) or blank (any). Distance reported as a fold change of medians where positive numbers mean more, negative numbers less sensitive.

Treatment	Reference	Comparison	p-value	FC (medians)
MAPK + Trametinib	MAPK_wt	MAPK_mut	4.65e-29	950
MAPK + Trametinib	MAPK-	MAPK+	8.91e-37	17930
MAPK + Trametinib	MAPK-_wt	MAPK+_wt	7.11e-22	1580
MAPK + Trametinib	MAPK-_mut	MAPK+_mut	7.13e-07	9130
MAPK + Trametinib	MAPK-_mut	MAPK+_wt	2.40e-02	15
MAPK + AZ628	MAPK_wt	MAPK_mut	1.03e-14	102
MAPK + AZ628	MAPK-	MAPK+	1.51e-11	2199
MAPK + AZ628	MAPK-_wt	MAPK+_wt	1.84e-04	20
MAPK + AZ628	MAPK-_mut	MAPK+_mut	1.41e-02	65
MAPK + AZ628	MAPK-_mut	MAPK+_wt	2.34e-02	-4
BRAF + Dabrafenib	BRAF_wt	BRAF_mut	1.88e-24	314085
BRAF + Dabrafenib	MAPK-	MAPK+	2.52e-09	14
BRAF + Dabrafenib	MAPK-_wt	MAPK+_wt	9.73e-04	4
BRAF + Dabrafenib	MAPK-_mut	MAPK+_mut	1.40e-01	54026
BRAF + Dabrafenib	MAPK-_mut	MAPK+_wt	3.64e-04	-33

Supplementary Table 6: Validation of significant GDSC associations in the CCLE with overlapping drugs (PD-0325901, AZD6244, and 17-AAG). Effect size is 10-fold drug response per pathway score standard deviation.

Dataset	Drug	Pathway	Effect size	FDR
CCLE	PD-0325901	MAPK	-1.16	1.6e-08
CCLE	PD-0325901	EGFR	-1.16	2.1e-07
CCLE	AZD6244	MAPK	-0.80	3.2e-05
CCLE	17-AAG	EGFR	-0.33	0.033
GDSC	PD-0325901	MAPK	-0.40	8e-05
GDSC	PD-0325901	EGFR	-0.35	0.0017
GDSC	17-AAG	EGFR	-0.29	0.026
GDSC	AZD6244	MAPK	-0.25	0.036

Supplementary Table 7: Stability of the highlighted survival associations across 100 bootstraps (resampling patients within each cohort with replacement). The associations are significant for 93 or more bootstraps and the median p-value is highliy significant.

Cohort	Pathway	Median p-value	# of significance (5% FDR)
ACC	p53	0.00012	98
KIRC	TNFa	6.1e-05	97
LGG	JAK-STAT	0.00029	93

Supplementary Figures

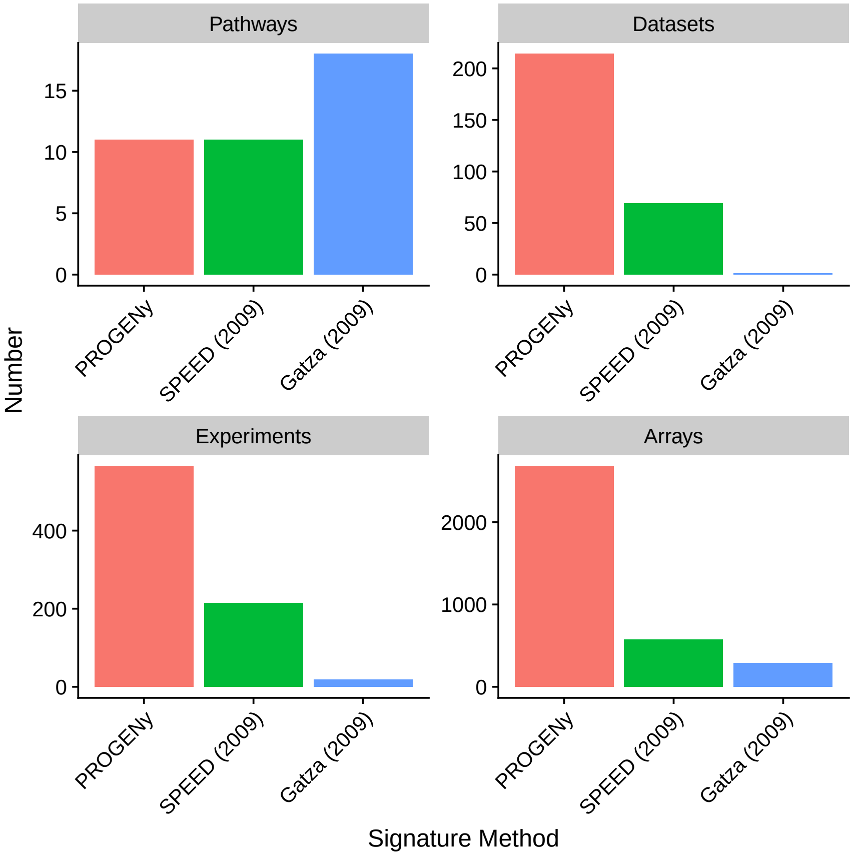

Supplementary Figure 1: Comparison of dataset size between SPEED (data source for Iorio 2016), Gatza (2009), and PROGENy. Gatza et al. derived 18 pathway signatures using only the MCF-10A cell line (thus also 18 experiments), and a total of 287 arrays. In 2014, they included additional signatures from other sources to a total of 53, but some are redundant, others not pathways, and all still limited to breast cancer. SPEED (Parikh et al. 2009) assembled consensus signatures for for 11 pathways using 69 GEO submissions, 215 different conditions and 572 arrays. Our data set consists of 11 pathways, 217 GEO submissions, 568 different experiments and a total of 2687 arrays. This means we use more evidence per pathway and cover a broader set of experimental conditions, but also reflects the imposed limitation of only considering experiments if there are at least two unperturbed arrays available in order to estimate the basal variability. In addition, all our expression values are derived from raw data and not preprocessed data that we can not reproduce.

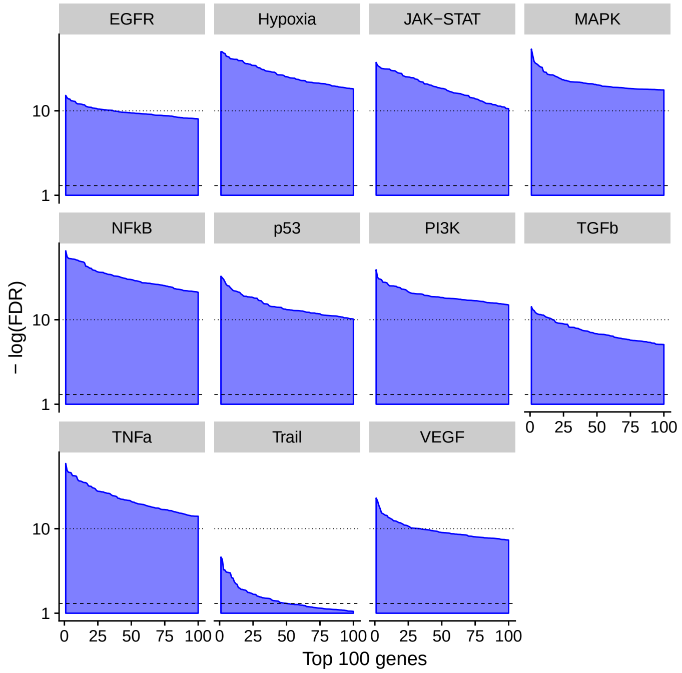

(a) Distribution of FDR-correctd p-values for signature genes (double log). Genes (horizonal axis) are ordered by significance. Dashed line at 5% FDR, dotted line at 10^{-10}

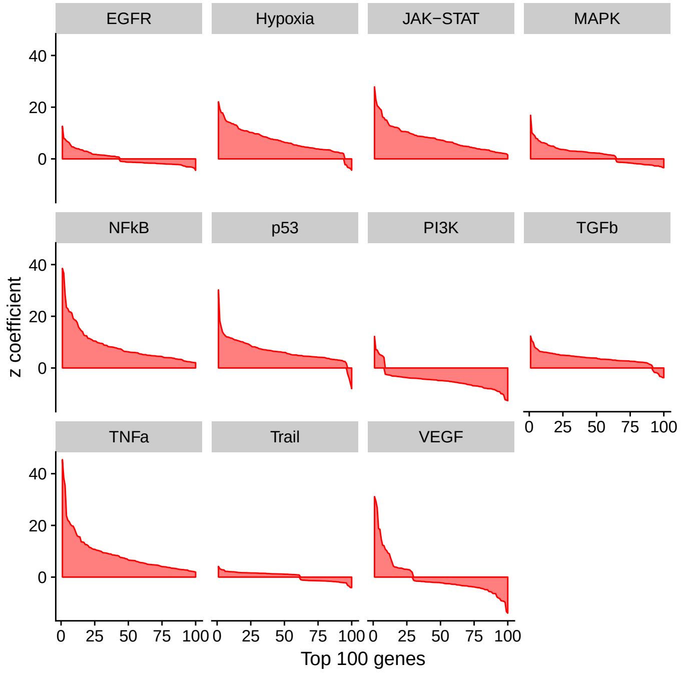

(b) Distribution of z-scores of the top 100 significant genes (different order compared to a). Signature genes are comprised of both up- and downregulated genes for most of the pathways. In no pathway there a single or a few z-scores are high enough to overshadow the rest of the signature, indicating that the model is numerically stable.

Supplementary Figure 2: Distribution of the top 100 genes used in the model, as outcome for the multiple regression of all pathways

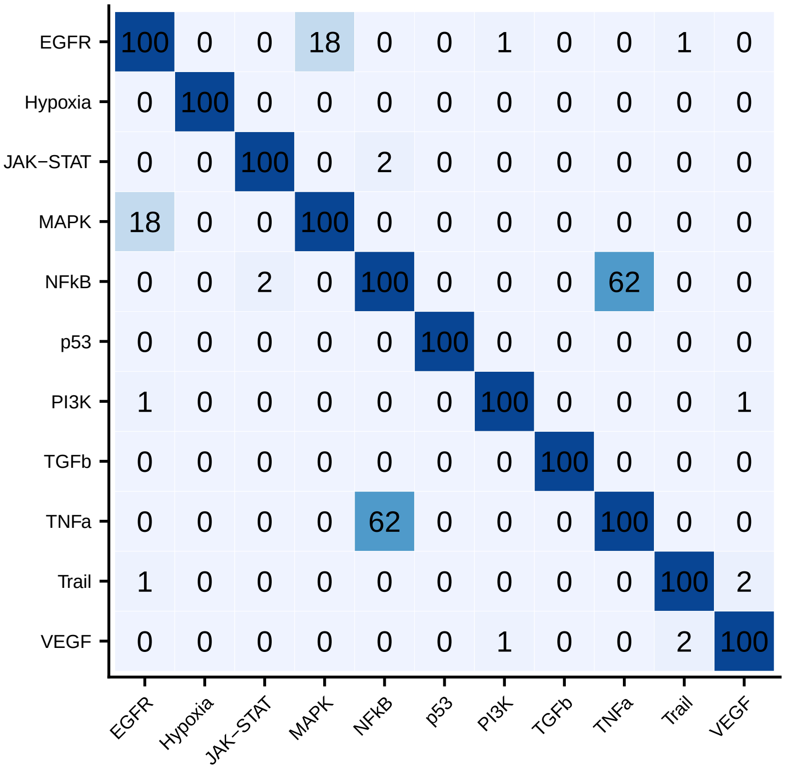

(a) Overlap of signature genes for different pathways. For the 100 genes we selected for each individual pathway, this shows how many of those signature genes are present in another pathway as well. The overlap is generally low, with the highest numbers between TNFa and NFkB (62) and EGFR and MAPK (18). As both of these pathway pairs have one component that is directly upstream of the other, this is to be exptected. This also shows that the response genes are specific to their perturbation and not a common phenotype like stress response because of any perturbation.

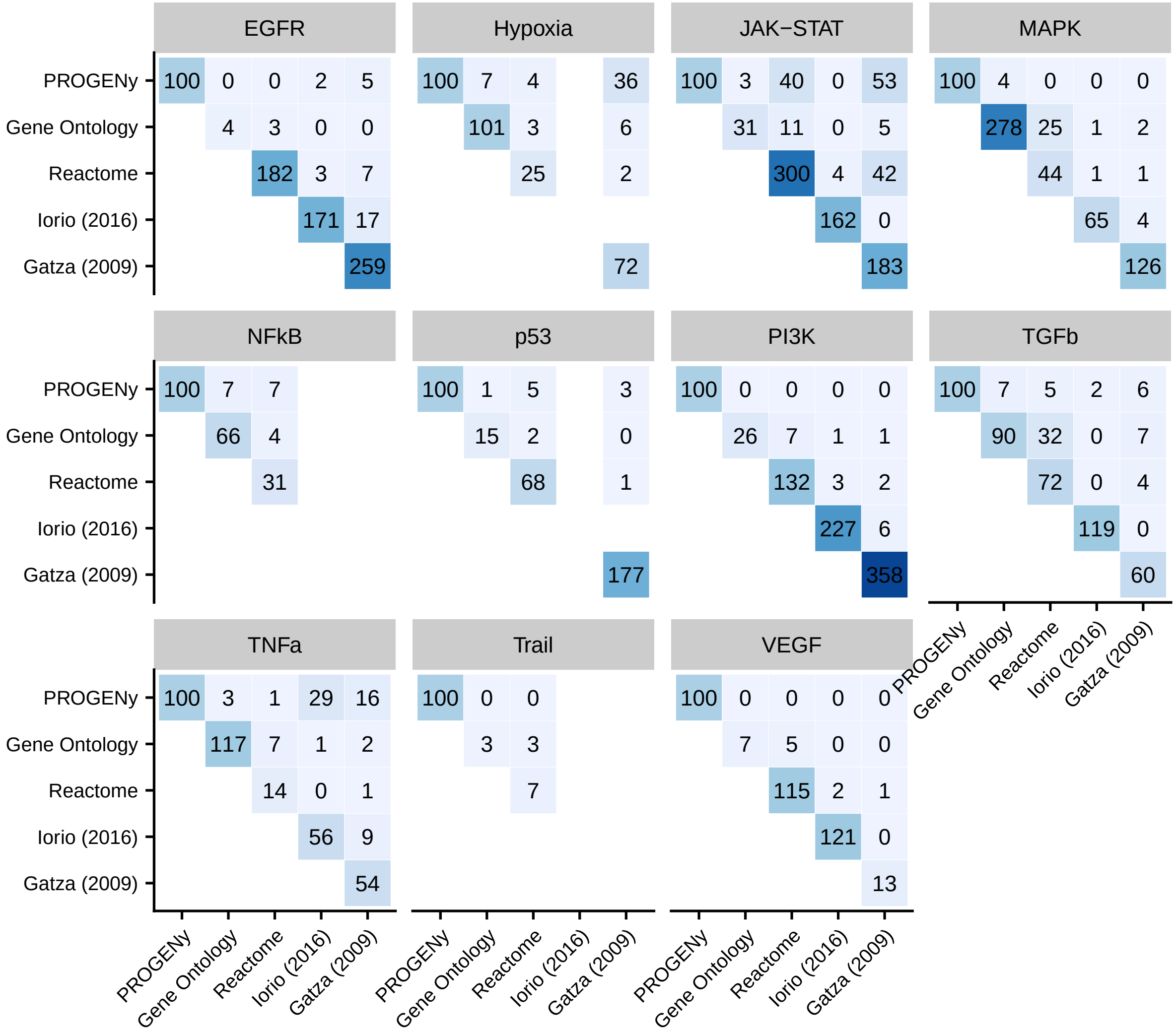

(b) Difference in gene sets between PROGENy genes and Gene Ontology, Reactome (incl. Pathifier), SPEED-derived Iorio (2016) and Gatza (2009). PROGENy genes are different to pathway members and gene annotations, with between 0 and 7 genes in common. Overlap with other signatures is higher, but still a minor fraction of the total number of signature genes.

Supplementary Figure 3: Overlap between different gene sets

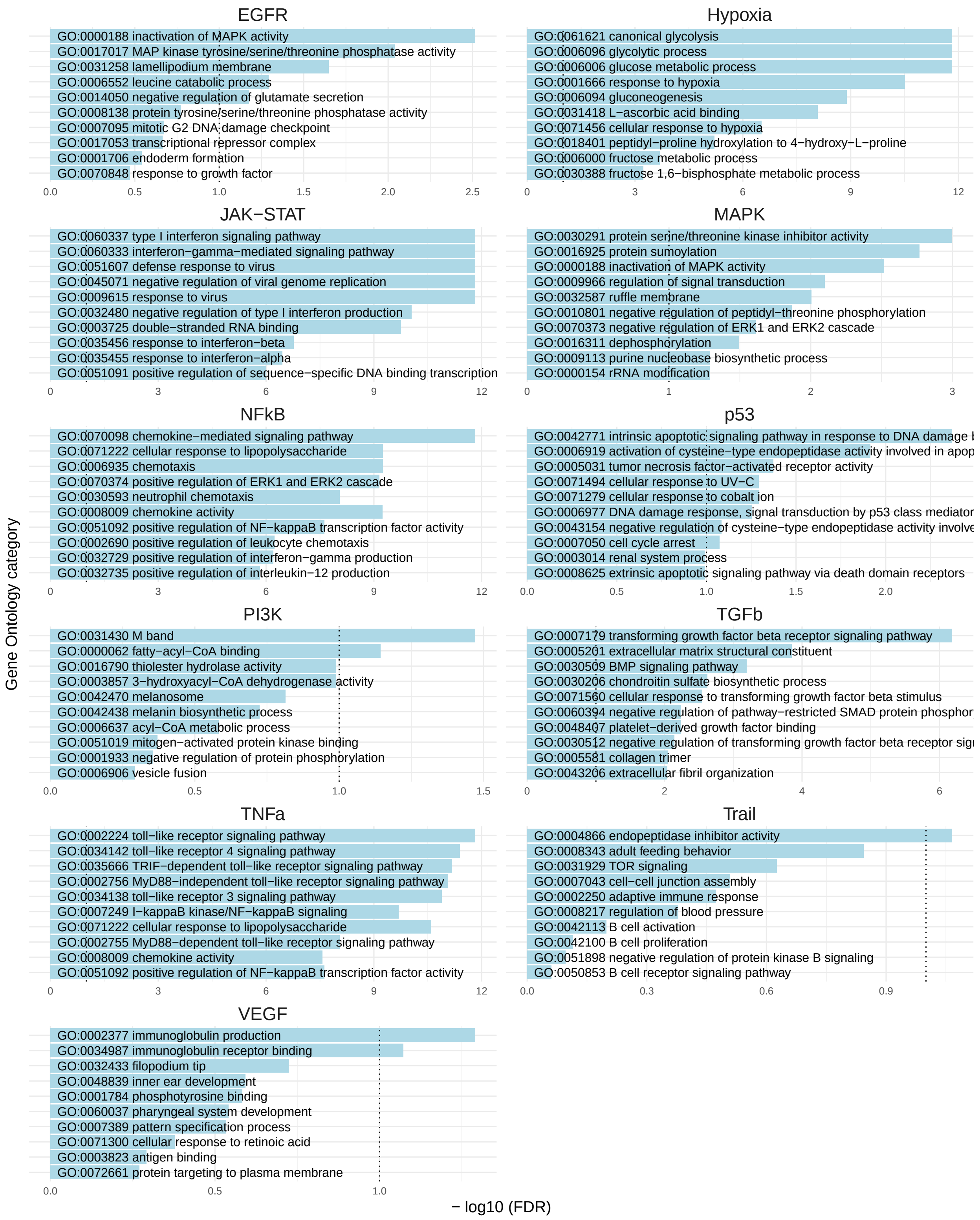

Supplementary Figure 4: Results of a hypergeometric test for enrichment in the overlap between the gene set derived in PROGENy and Gene Ontology categories between 5 and 200 genes in size. Each panel corresponds to one pathway, length of the bars to the negative log10 of the FDR. Top 10 categories are shown for each pathway. Dotted vertical line represents 10% FDR.

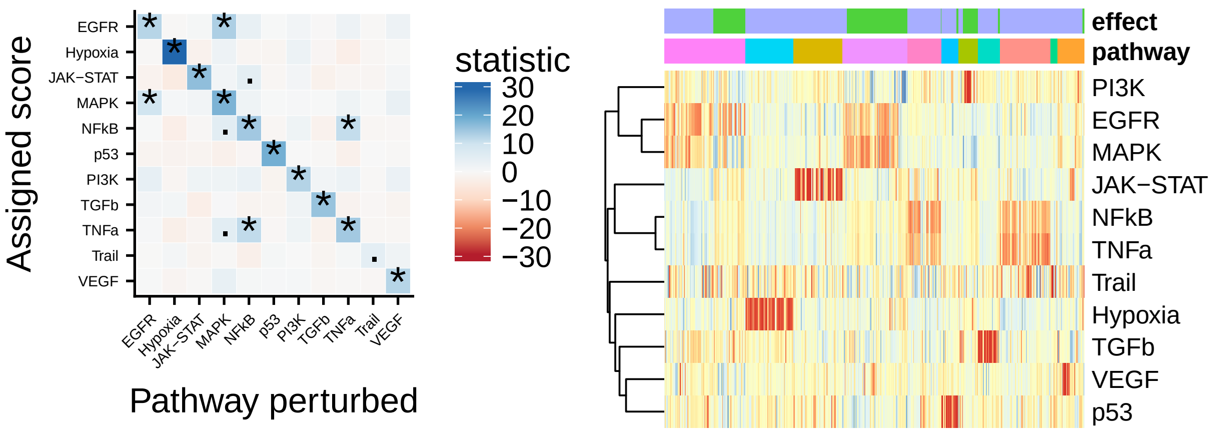

(a) Perturbation-response genes

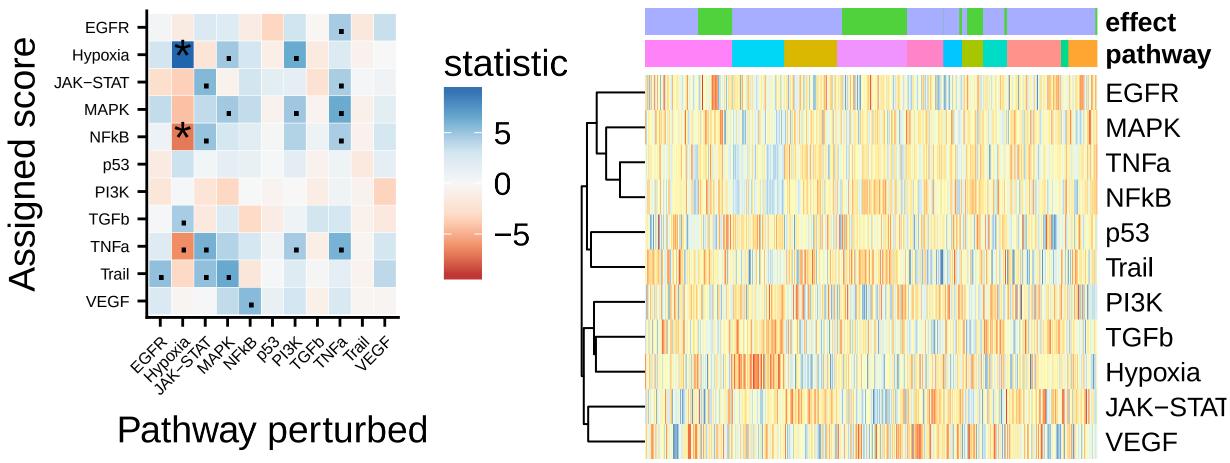

(b) Gene Ontology

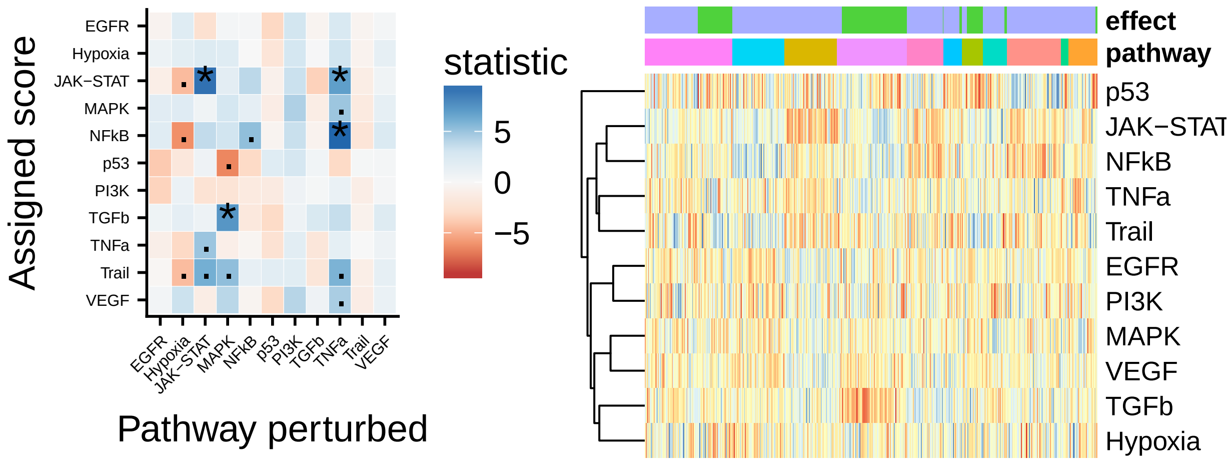

(c) Reactome

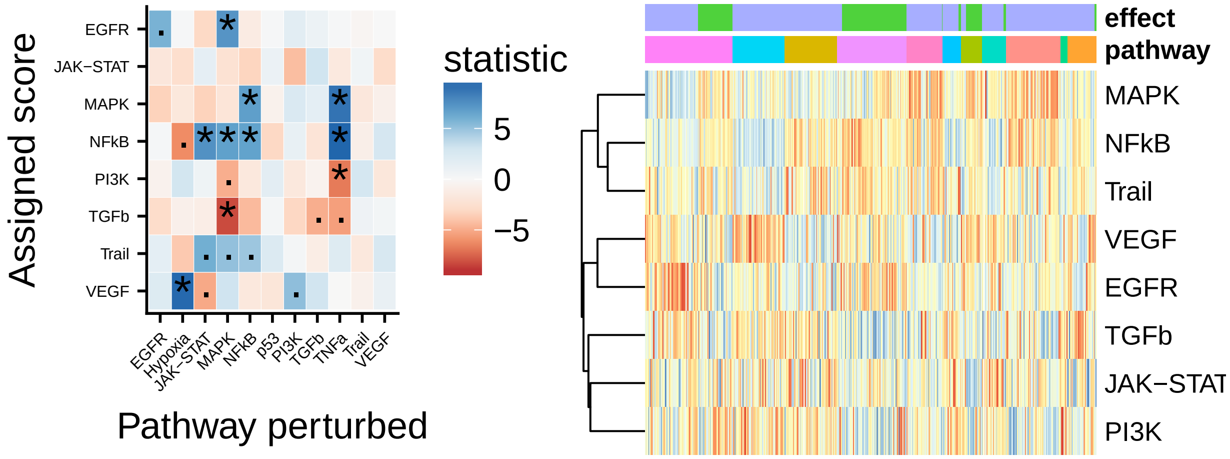

(d) SPIA

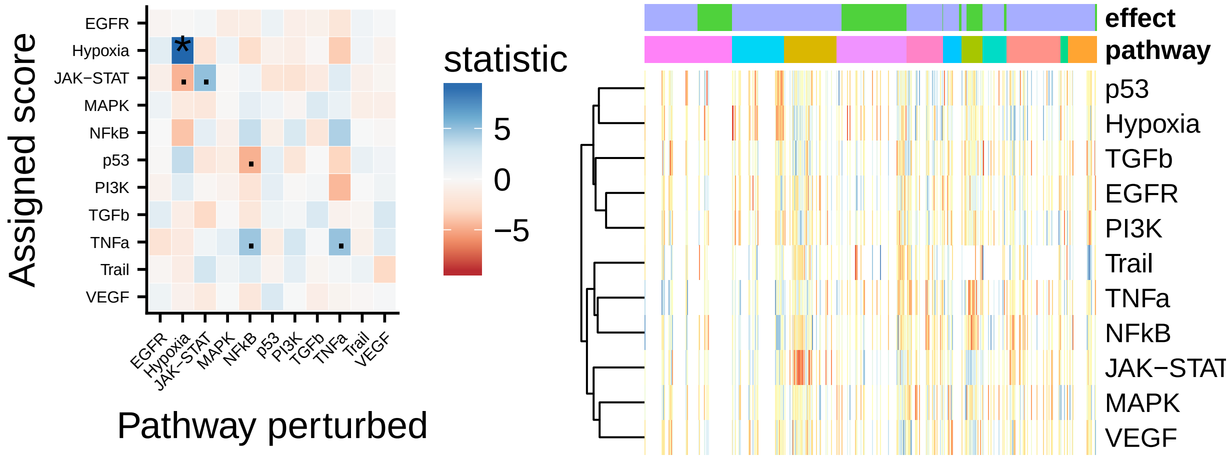

(e) Pathifier

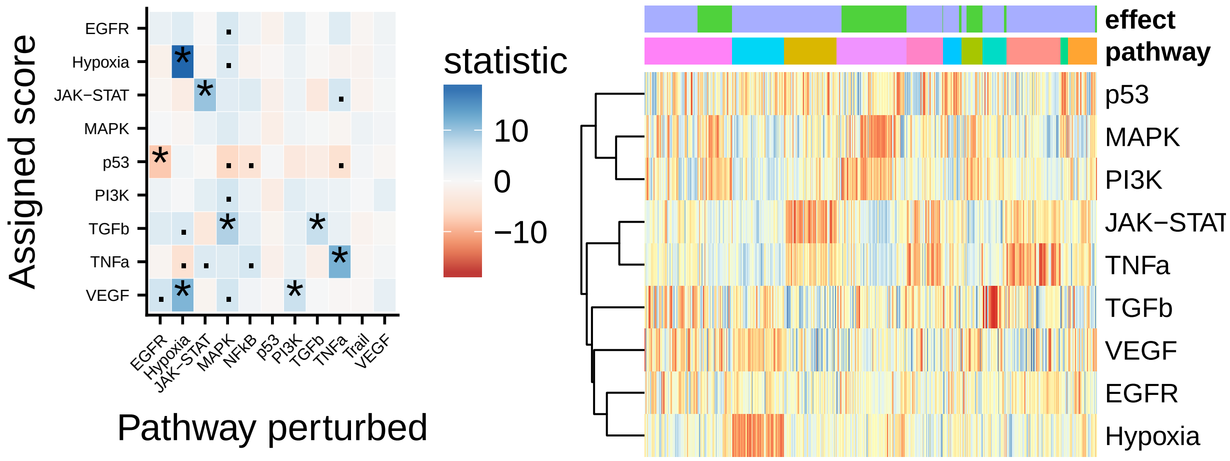

(f) Gatza (2009)

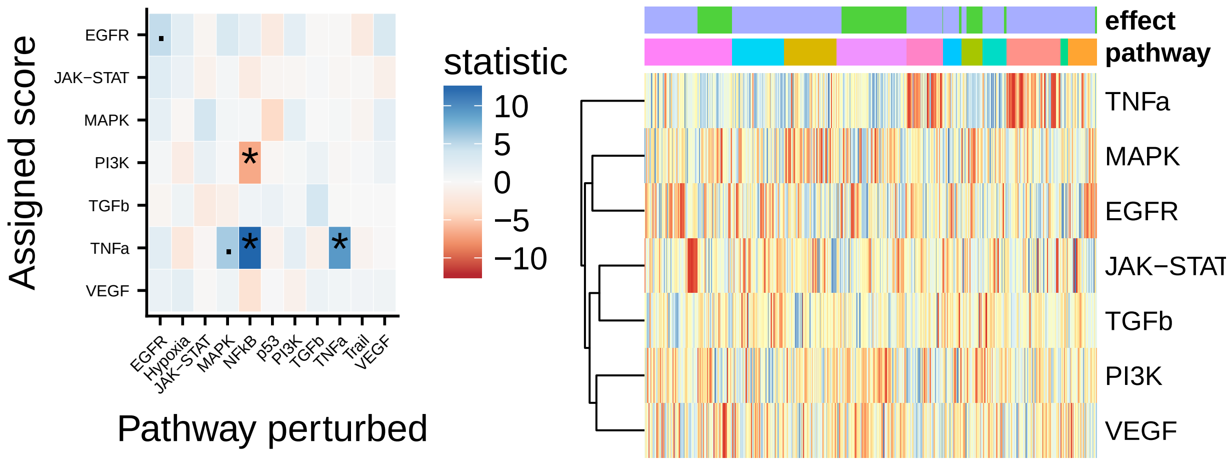

(g) SPEED-derived (Iorio et al., 2016)

Supplementary Figure 5: Distribution of pathway scores for individual experiments (right) and their statistics (left). Right: perturbation experiments on horizontal axis, ordered alphabetically as indicated by color bar for pathways. Effect of a perturbatin on the corresponding pathway is activating (teal) or inhibiting (orange). Cells in the heatmap correspond to relative pathway activation according to pathway scores for different methods. Pathway inhibitions shown in the activating direction (negative inhibition). White cells indicate experiments where we could not derive pathway scores (Pathifier). Left: statistical associations between perturbed pathway and change in scores for all pathway scores given any perturbation. Significance indicated with a dot for $FDR < 10^{-5}$ and a star for $FDR < 10^{-10}$, color grading by Wald statistic. For perturbation-response genes, pathway scores (rows in heatmap) cluster for EGFR/MAPK and to a lesser extent PI3K, as well as TNFa/NFkB, supported by statistical associations. As this was performed on the same set of experiments as was used to create the model, these associations only quantify the amount of cross-activation and heterogeneity in the response, the significance of the model itself is shown in figure 2. It does, however, show that competing pathway methods are for the most part unable to recover perturbed pathways or associate with a different perturbation than the one applied. This highlights that methods based on pathway mapping should only interpreted as either only expression level instead of activity, or potential signal flow as opposed to actual signal flow.

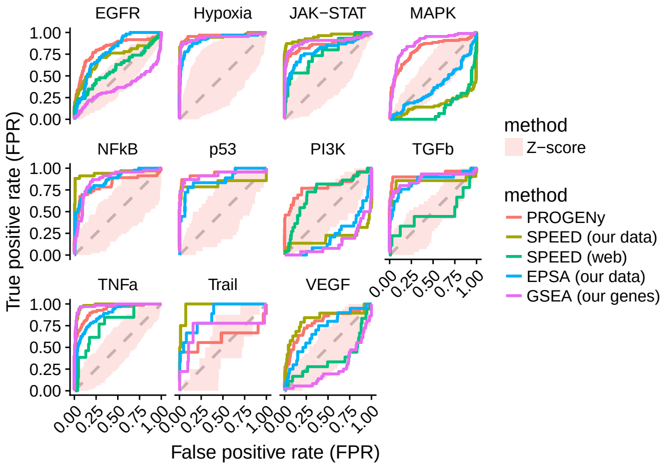

(a) Comparison between PROGENy (linear model of z-scores), SPEED (Fisher's Exact Test for z-scores, where web refers to the web tool at http://speed.sys-bio.net and our data to using an FET on our curated experiments), and EPSA (Spearman correlation between significant median fold changes of the model and the fold changes of each perturbation experiment). Additionally, GSEA (KS-test) using the PROGENy gene list and the highest z-scores of individual experiments (red shade) are shown.

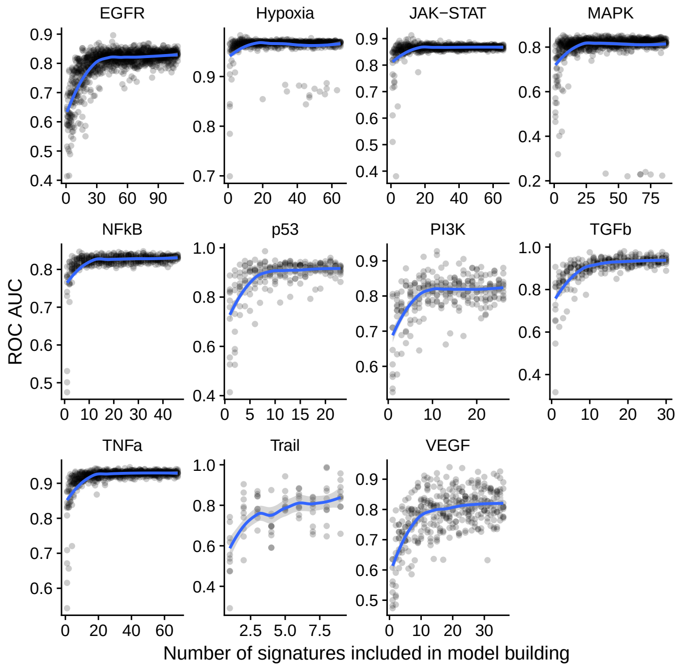

(b) AUC with different number of signatures. The PROGENy model is build limiting each pathway to n signatures. The obtained AUC first increases quickly with few signatures and then levels off between 10 and 40 signatures.

Supplementary Figure 6: ROC curves for different ways to compute consensus models (a) and smaller sets of signatures (b). For each signature, we test if perturbed pathways are ordered before non-perturbed pathways (negative fold changes taken for inhibitions)

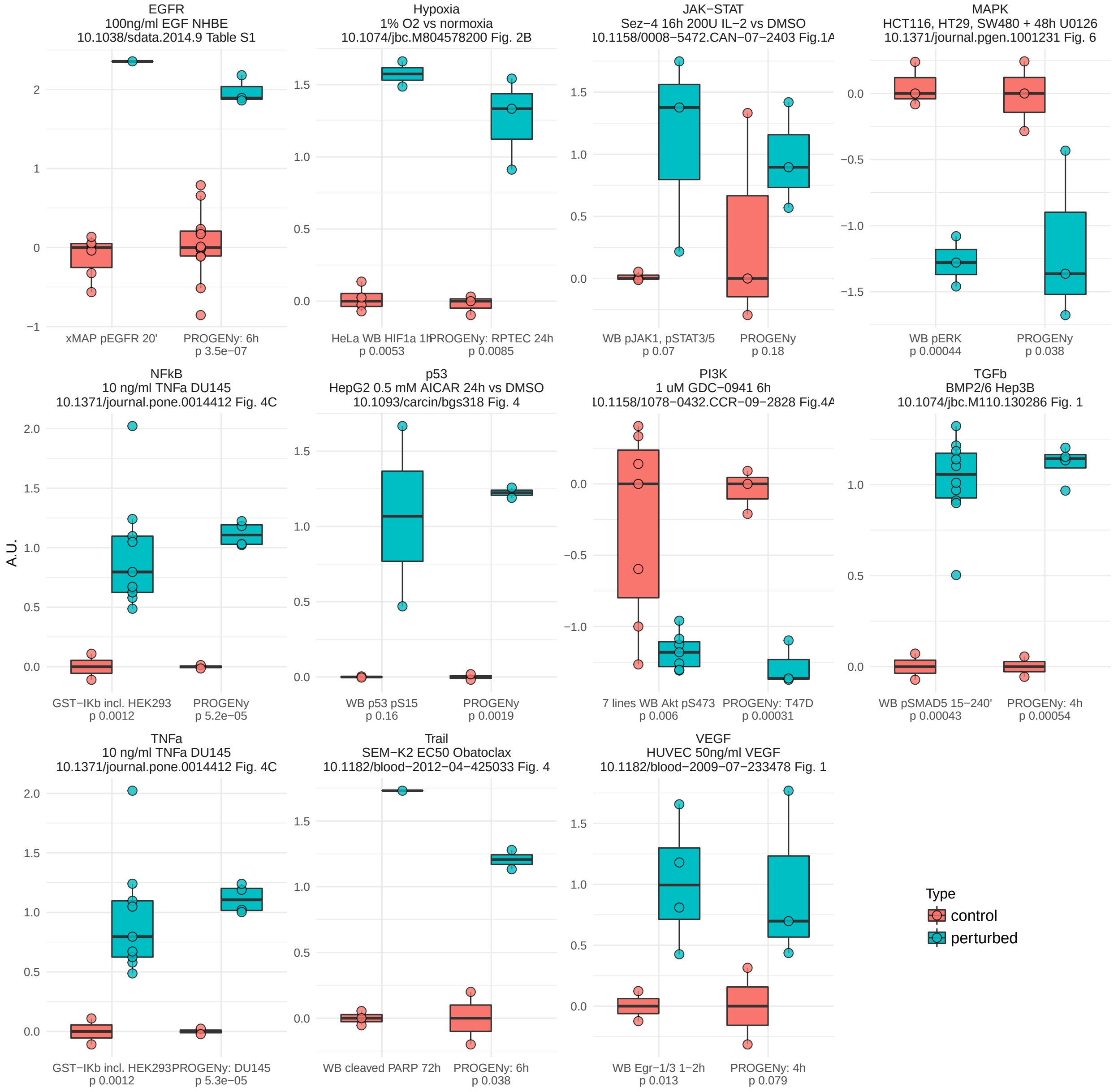

Supplementary Figure 7: Relative pathway activation patterns for activity measurement vs. inference by PROGENy. Data sources (publication DOI in panel title) are experiments where PTMs (mostly Western Blots of pathway drivers) and gene expression were measured in at least duplicate and that were not previously included in model building. Each panel shows a pathway with activity measurement on the left and inferred pathway score on the right. The Y-axis is scaled to a normal distribution and should be used only to compare basal vs. perturbed experiments of the same kind. Difference quantified using a one-tailed t-test. Hinges represent quartiles, whiskers 1.5*IQR.

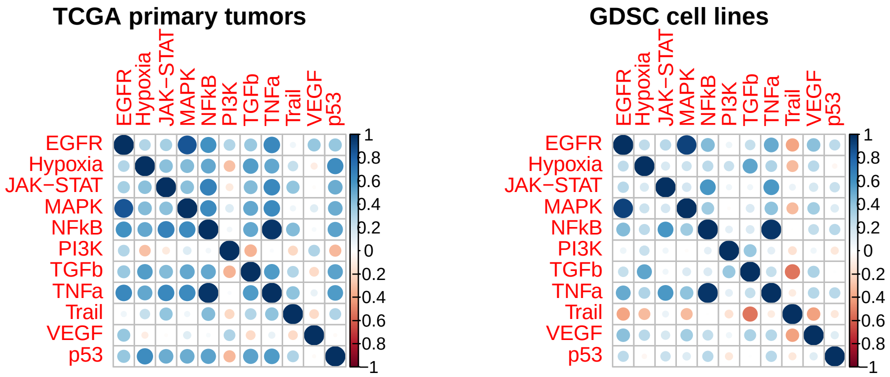

(a) Perturbation-response genes

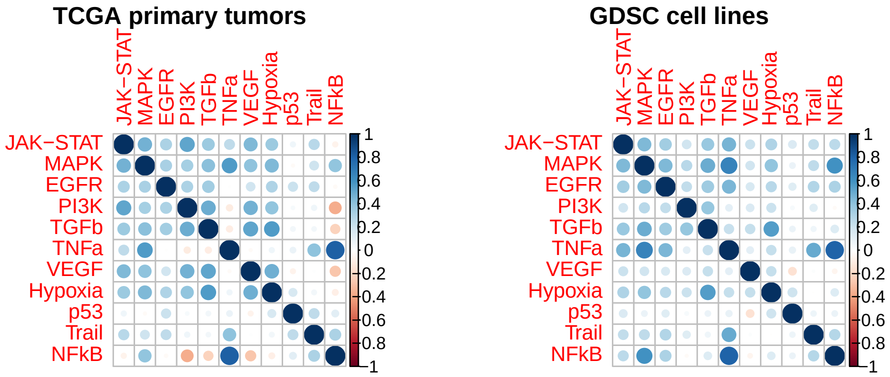

(b) Gene Ontology

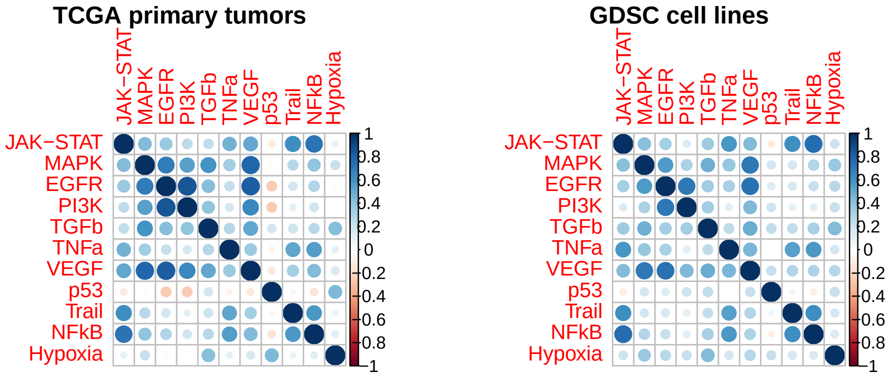

(c) Reactome

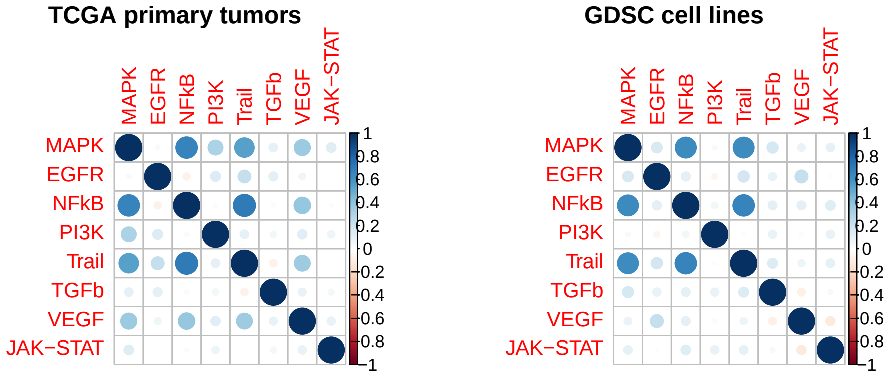

(d) SPIA

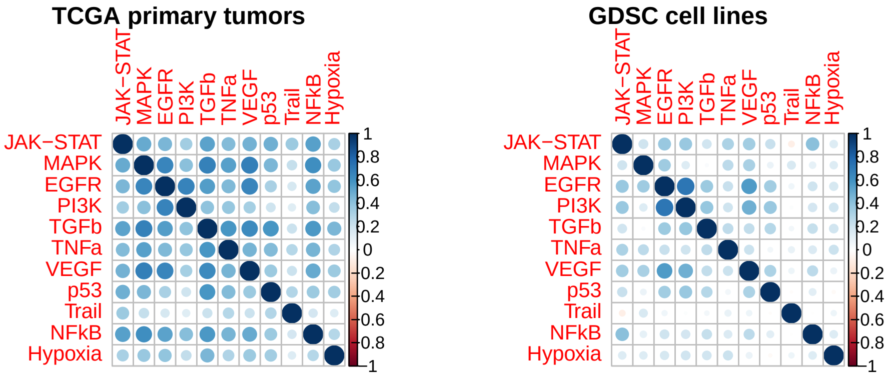

(e) Pathifier

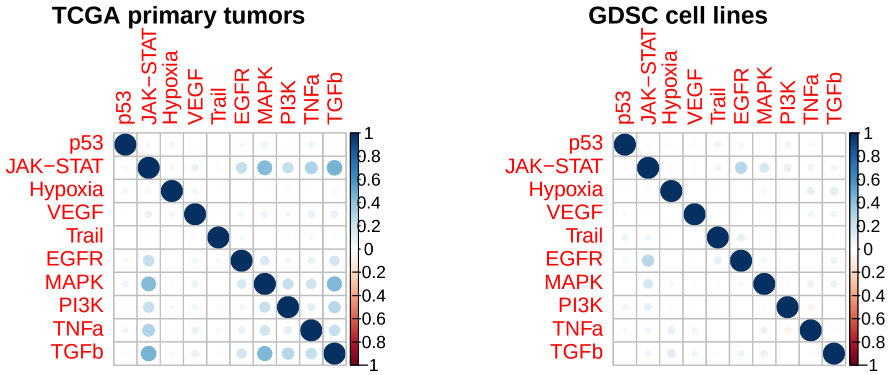

(f) PARADIGM

Supplementary Figure 8: Correlation of pathway scores in basal expression of primary tumors in the TCGA (left) and cell lines in the GDSC (right). Perturbation-response genes show similar correlation in basal correlation to cross-activation in the perturbation experiments. Gene Ontology, Reactome and Biocarta show a higher correlation overall, hinting at the fact that transcription of related pathways is less heterogeneous than the post-translational activity they mediate. For those methods, correlations within tumors vs. cell lines look similar. SPIA and Pathifier are less comparable between TCGA and GDSC because these methods require comparison to a reference condition (Tumor normals in the TCGA) that we could only provide by comparing each cell line in the GDSC to the rest of cell lines in that tissue. This makes those correlations less comparable between the two panels for each method. Apart from that, Pathifier shows very high correlation if computing scores against normals, suggesting that this method may not be suitable to differentiate between pathways using TCGA normals. PARADIGM shows almost no correlation between pathways.

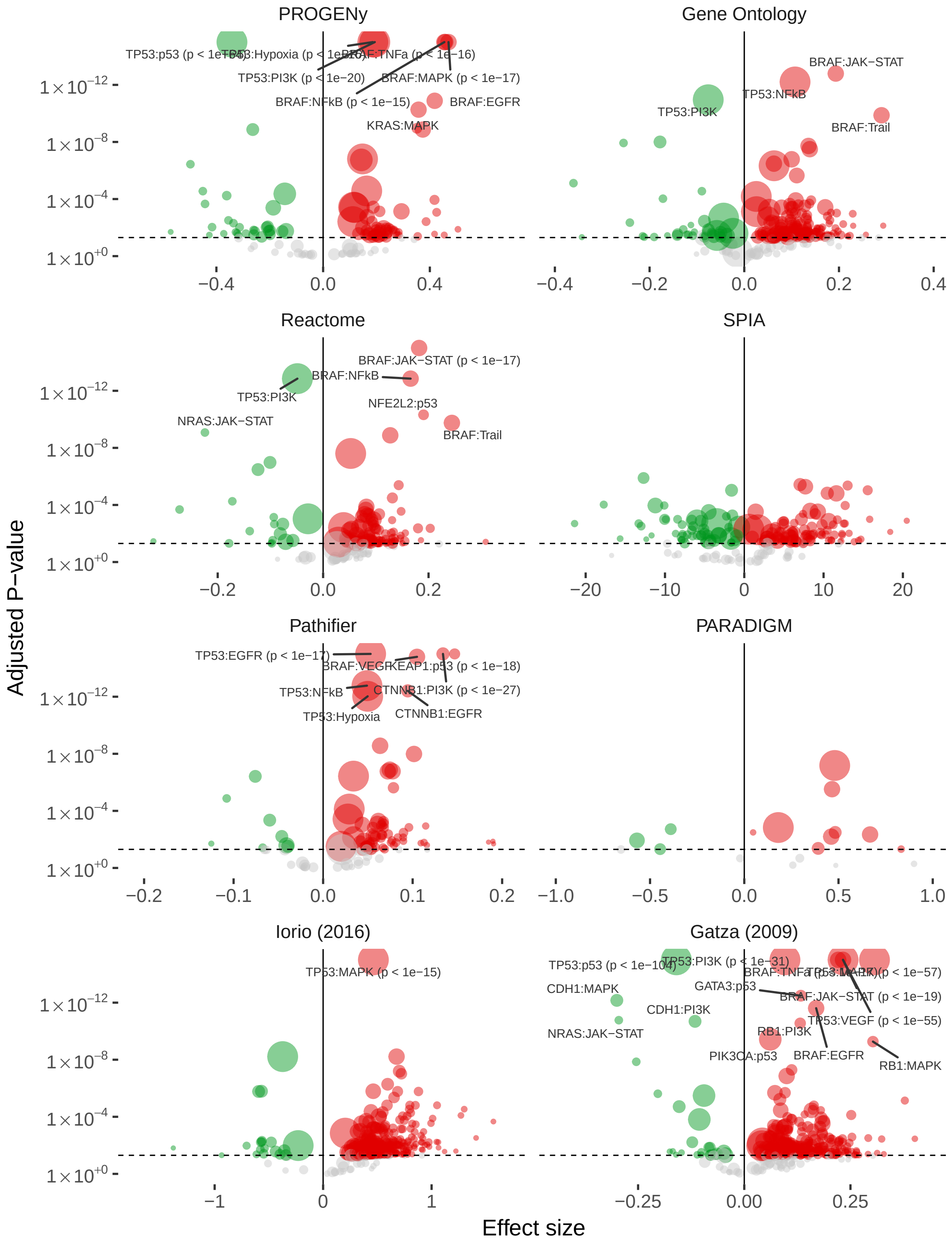

Supplementary Figure 9: Volcano plot for associations between pathway scores and mutated driver genes. Effect size is standard deviations of pathway scores. P-values FDR-corrected. Associations corrected for cancer type. PROGENy provides stronger associations and are more in line with literature knowledge of signaling pathways.

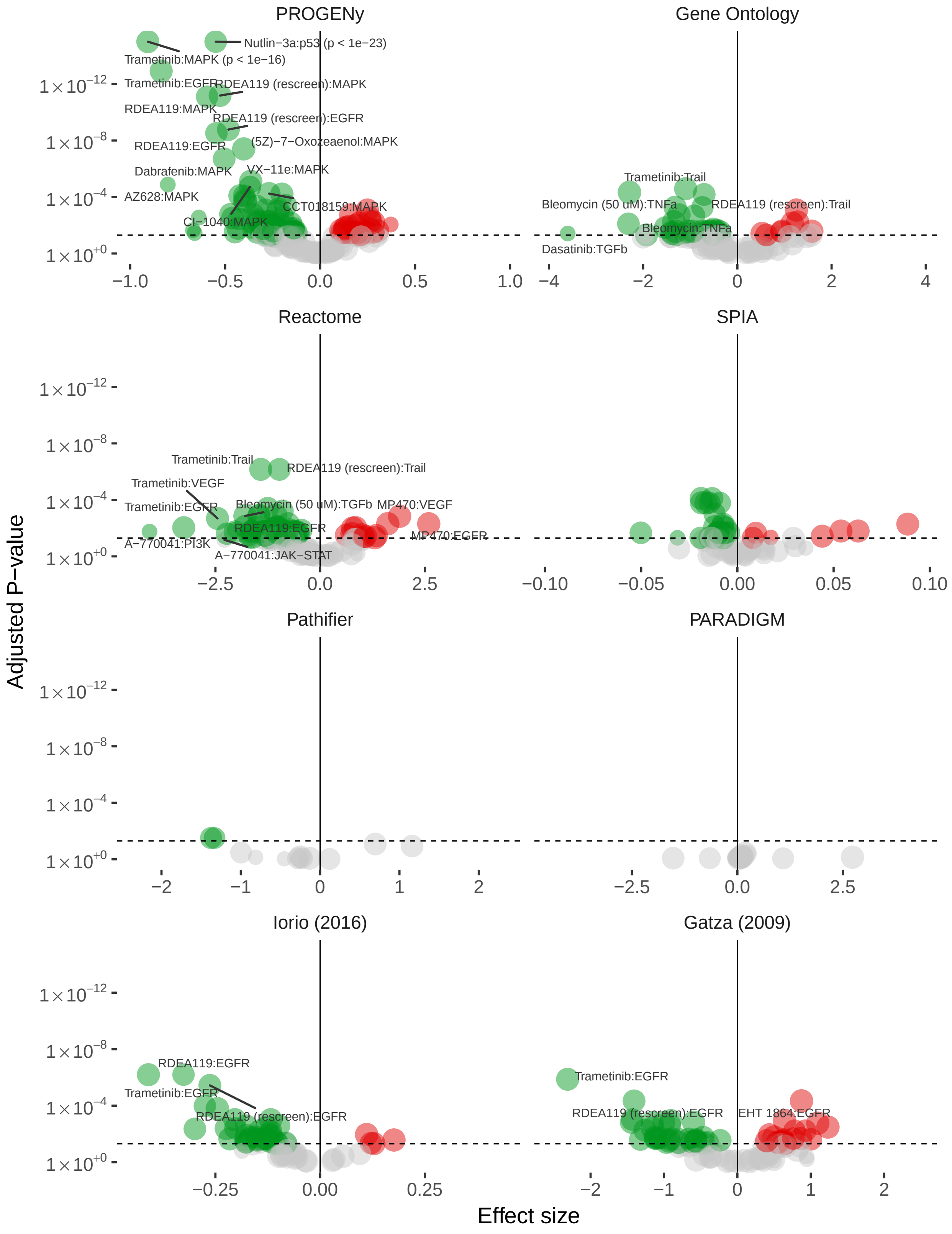

Supplementary Figure 10: Volcano plots for associations between pathway scores and drug response (IC50). Effect size arbitrary units, p-values FDR-corrected. Pathway-response genes are the only method to recover highly significant oncogene addiction associations, the rest of methods show no obvious connection between the drug target and pathway.

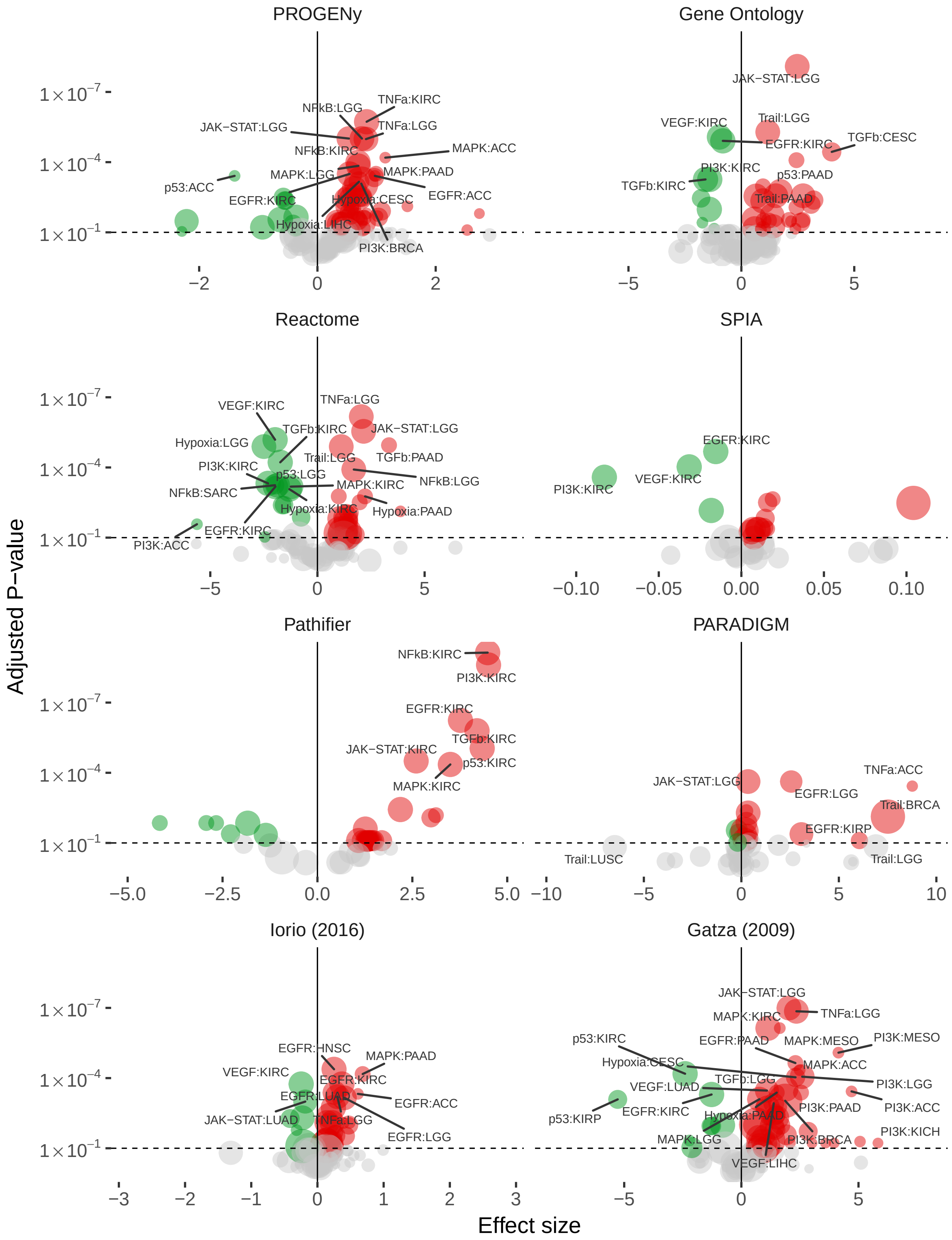

Supplementary Figure 11: Volcano plots for tissue-specific survival associations. Effect size arbitrary units, p-values FDR-corrected. All methods show strongest associations with KIRC and LGG. Pathway-response genes only method to separate associations into classical oncogenic and tumor suppressor pathways, calling into question the meaning of associations obtained by mapping gene expression on pathway components.

Supplementary References

1. Wujcik, D. EGFR as a Target: Rationale for Therapy. *Seminars in Oncology Nursing* **22,** 5–9 (Feb. 2006).
2. Schneider, M. R. & Yarden, Y. Structure and function of epigen, the last EGFR ligand. *Seminars in Cell & Developmental Biology* **28,** 57–61 (Apr. 2014).
3. Johnston, J. *et al.* Targeting the EGFR Pathway for Cancer Therapy. *Current Medicinal Chemistry* **13,** 3483–3492 (Dec. 2006).
4. Ono, M. & Kuwano, M. Molecular Mechanisms of Epidermal Growth Factor Receptor (EGFR) Activation and Response to Gefitinib and Other EGFR-Targeting Drugs. *Clinical Cancer Research* **12,** 7242–7251 (Dec. 2006).
5. Ellis, A. *et al.* High-performance liquid chromatographic analysis of the tyrphostin AG1478, a specific inhibitor of the epidermal growth factor receptor tyrosine kinase, in mouse plasma. *Journal of Chromatography B: Biomedical Sciences and Applications* **754,** 193–199 (Apr. 2001).
6. Misior, A. M. *et al.* Glucocorticoid- and Protein Kinase A–Dependent Transcriptome Regulation in Airway Smooth Muscle. *American Journal of Respiratory Cell and Molecular Biology* **41,** 24–39 (July 2009).
7. Essaghir, A., Dif, N., Marbehant, C. Y., Coffer, P. J. & Demoulin, J.-B. The Transcription of FOXO Genes Is Stimulated by FOXO3 and Repressed by Growth Factors. *Journal of Biological Chemistry* **284,** 10334–10342 (Feb. 2009).
8. Wolfer, A. *et al.* MYC regulation of a "poor-prognosis" metastatic cancer cell state. *Proceedings of the National Academy of Sciences* **107,** 3698–3703 (Feb. 2010).
9. Liu, L. *et al.* Novel Mechanism of Lapatinib Resistance in HER2-Positive Breast Tumor Cells: Activation of AXL. *Cancer Research* **69,** 6871–6878 (Aug. 2009).
10. van Leenders, G. J. L. H. *et al.* Activation of c-MET Induces a Stem-Like Phenotype in Human Prostate Cancer. *PLoS ONE* **6** (ed Sarkar, F. H.) e26753 (Nov. 2011).
11. Chan, G., Nogalski, M. T. & Yurochko, A. D. Activation of EGFR on monocytes is required for human cytomegalovirus entry and mediates cellular motility. *Proceedings of the National Academy of Sciences* **106,** 22369–22374 (Dec. 2009).
12. Jürchott, K. *et al.* Identification of Y-Box Binding Protein 1 As a Core Regulator of MEK/ERK Pathway-Dependent Gene Signatures in Colorectal Cancer Cells. *PLoS Genetics* **6** (ed Cheung, V. G.) e1001231 (Dec. 2010).
13. Oyama, M. *et al.* Integrated Quantitative Analysis of the Phosphoproteome and Transcriptome in Tamoxifen-resistant Breast Cancer. *Journal of Biological Chemistry* **286,** 818–829 (Nov. 2010).
14. Ghosh, R. *et al.* Trastuzumab Has Preferential Activity against Breast Cancers Driven by HER2 Homodimers. *Cancer Research* **71,** 1871–1882 (Feb. 2011).
15. Isella, C., Renzulli, T., à, D. C. & Medico, E. Mulcom: a multiple comparison statistical test for microarray data in Bioconductor. *BMC Bioinformatics* **12,** 382 (2011).
16. Lee, M. *et al.* Sequential Application of Anticancer Drugs Enhances Cell Death by Rewiring Apoptotic Signaling Networks. *Cell* **149,** 780–794 (May 2012).
17. Tran, Q. T. *et al.* EGFR regulation of epidermal barrier function. *Physiological Genomics* **44,** 455–469 (Mar. 2012).
18. Fertig, E. J. *et al.* Gene expression signatures modulated by epidermal growth factor receptor activation and their relationship to cetuximab resistance in head and neck squamous cell carcinoma. *BMC Genomics* **13,** 160 (2012).
19. Li, M. *et al.* Guanylate binding protein 1 is a novel effector of EGFR-driven invasion in glioblastoma. *The Journal of Experimental Medicine* **208,** 2657–2673 (Dec. 2011).

20. Byun, J. S. *et al.* ELL facilitates RNA polymerase II pause site entry and release. *Nature Communications* **3,** 633 (Jan. 2012).

21. Toriseva, M. *et al.* Keratinocyte Growth Factor Induces Gene Expression Signature Associated with Suppression of Malignant Phenotype of Cutaneous Squamous Carcinoma Cells. *PLoS ONE* **7** (ed Brandner, J. M.) e33041 (Mar. 2012).

22. Alajati, A. *et al.* Mammary Tumor Formation and Metastasis Evoked by a HER2 Splice Variant. *Cancer Research* **73,** 5320–5327 (July 2013).

23. Rothenberg, S. M. *et al.* Inhibition of mutant EGFR in lung cancer cells triggers SOX2-FOXO6-dependent survival pathways. *eLife* **4.** doi:`10.7554/elife.06132`. `https://doi.org/10.7554/elife.06132` (Feb. 2015).

24. Waters, K. M., Cummings, B. S., Shankaran, H., Scholpa, N. E. & Weber, T. J. ERK Oscillation-Dependent Gene Expression Patterns and Deregulation by Stress Response. *Chemical Research in Toxicology* **27,** 1496–1503 (Sept. 2014).

25. Lee, H.-J. *et al.* Drug Resistance via Feedback Activation of Stat3 in Oncogene-Addicted Cancer Cells. *Cancer Cell* **26,** 207–221 (Aug. 2014).

26. Semenza, G. L. Hypoxia-inducible factor 1: master regulator of O2 homeostasis. *Current Opinion in Genetics & Development* **8,** 588–594 (Oct. 1998).

27. Gordan, J. D. *et al.* HIF- Effects on c-Myc Distinguish Two Subtypes of Sporadic VHL-Deficient Clear Cell Renal Carcinoma. *Cancer Cell* **14,** 435–446 (Dec. 2008).

28. Fardin, P. *et al.* The l1-l2 regularization framework unmasks the hypoxia signature hidden in the transcriptome of a set of heterogeneous neuroblastoma cell lines. *BMC Genomics* **10,** 474 (2009).

29. Lee, J. J. *et al.* Hypoxia activates the cyclooxygenase-2–prostaglandin E synthase axis. *Carcinogenesis* **31,** 427–434 (Dec. 2009).

30. Fardin, P. *et al.* A biology-driven approach identifies the hypoxia gene signature as a predictor of the outcome of neuroblastoma patients. *Molecular Cancer* **9,** 185 (2010).

31. Xia, X. & Kung, A. L. Preferential binding of HIF-1 to transcriptionally active loci determines cell-type specific response to hypoxia. *Genome Biology* **10,** R113 (2009).

32. Aryee, D. N. T. *et al.* Hypoxia Modulates EWS-FLI1 Transcriptional Signature and Enhances the Malignant Properties of Ewing's Sarcoma Cells In vitro. *Cancer Research* **70,** 4015–4023 (May 2010).

33. Bosco, M. C. *et al.* Hypoxia modulates the gene expression profile of immunoregulatory receptors in human mature dendritic cells: identification of TREM-1 as a novel hypoxic marker in vitro and in vivo. *Blood* **117,** 2625–2639 (Dec. 2010).

34. Kirwan, R., Felice, L., Clark, A. F., O, C. J. & Leonard, M. O. Hypoxia Regulated Gene Transcription in Human Optic Nerve Lamina Cribrosa Cells in Culture. *Investigative Opthalmology & Visual Science* **53,** 2243 (Apr. 2012).

35. Koh, M. Y., Lemos, R., Liu, X. & Powis, G. The Hypoxia-Associated Factor Switches Cells from HIF-1 - to HIF-2 -Dependent Signaling Promoting Stem Cell Characteristics, Aggressive Tumor Growth and Invasion. *Cancer Research* **71,** 4015–4027 (Apr. 2011).

36. Tang, X. *et al.* Functional Interaction between Responses to Lactic Acidosis and Hypoxia Regulates Genomic Transcriptional Outputs. *Cancer Research* **72,** 491–502 (Dec. 2011).

37. Leithner, K. *et al.* Hypoxia increases membrane metallo-endopeptidase expression in a novel lung cancer ex vivo model – role of tumor stroma cells. *BMC Cancer* **14.** doi:`10.1186/1471-2407-14-40`. `https://doi.org/10.1186/1471-2407-14-40` (Jan. 2014).

38. Nissou, M.-F. *et al.* Hypoxia-induced expression of VE-cadherin and filamin B in glioma cell cultures and pseudopalisade structures. *Journal of Neuro-Oncology* **113,** 239–249 (Mar. 2013).

39. Das, A. M. *et al.* Melanomas prevent endothelial cell death under restrictive culture conditions by signaling through AKT and p38 MAPK/ ERK-1/2 cascades. *OncoImmunology* **5,** e1219826 (Aug. 2016).

40. Flamant, L. *et al.* TMEM45A is essential for hypoxia-induced chemoresistance in breast and liver cancer cells. *BMC Cancer* **12.** doi:10.1186/1471-2407-12-391. https://doi.org/10.1186/1471-2407-12-391 (Sept. 2012).

41. Kim, J.-w., Tchernyshyov, I., Semenza, G. L. & Dang, C. V. HIF-1-mediated expression of pyruvate dehydrogenase kinase: A metabolic switch required for cellular adaptation to hypoxia. *Cell Metabolism* **3,** 177–185 (Mar. 2006).

42. Onnis, B., Fer, N., Rapisarda, A., Perez, V. S. & Melillo, G. Autocrine production of IL-11 mediates tumorigenicity in hypoxic cancer cells. *Journal of Clinical Investigation* **123,** 1615–1629 (Mar. 2013).

43. Kushal, S. *et al.* Protein domain mimetics as in vivo modulators of hypoxia-inducible factor signaling. *Proceedings of the National Academy of Sciences* **110,** 15602–15607 (Sept. 2013).

44. Maiso, P. *et al.* Metabolic Signature Identifies Novel Targets for Drug Resistance in Multiple Myeloma. *Cancer Research* **75,** 2071–2082 (Mar. 2015).

45. Olbryt, M. *et al.* Global Gene Expression Profiling in Three Tumor Cell Lines Subjected to Experimental Cycling and Chronic Hypoxia. *PLoS ONE* **9** (ed Koritzinsky, M.) e105104 (Aug. 2014).

46. Sena, J. A., Wang, L., Heasley, L. E. & Hu, C.-J. Hypoxia Regulates Alternative Splicing of HIF and non-HIF Target Genes. *Molecular Cancer Research* **12,** 1233–1243 (May 2014).

47. Skowronki, K., Andrews, J., Rodenhiser, D. I. & Coomber, B. L. Genome-Wide Analysis in Human Colorectal Cancer Cells Reveals Ischemia-Mediated Expression of Motility Genes via DNA Hypomethylation. *PLoS ONE* **9** (ed Brim, H.) e103243 (July 2014).

48. Leisz, S. *et al.* Distinct von Hippel-Lindau gene and hypoxia-regulated alterations in gene and protein expression patterns of renal cell carcinoma and their effects on metabolism. *Oncotarget* **6,** 11395–11406 (Mar. 2015).

49. Chen, J. L.-Y. *et al.* The Genomic Analysis of Lactic Acidosis and Acidosis Response in Human Cancers. *PLoS Genetics* **4** (ed Gibson, G.) e1000293 (Dec. 2008).

50. Darnell, J., Kerr, I. & Stark, G. Jak-STAT pathways and transcriptional activation in response to IFNs and other extracellular signaling proteins. *Science* **264,** 1415–1421 (June 1994).

51. Schindler, C., Levy, D. E. & Decker, T. JAK-STAT Signaling: From Interferons to Cytokines. *Journal of Biological Chemistry* **282,** 20059–20063 (May 2007).

52. HEINRICH, P. C., BEHRMANN, I., MÜLLER-NEWEN, G., SCHAPER, F. & GRAEVE, L. Interleukin-6-type cytokine signalling through the gp130/Jak/STAT pathway. *Biochemical Journal* **334,** 297–314 (Sept. 1998).

53. Mortensen, O. H. *et al.* Calprotectin is released from human skeletal muscle tissue during exercise. *The Journal of Physiology* **586,** 3551–3562 (July 2008).

54. Stegmann, K. A. *et al.* Interferon-–Induced TRAIL on Natural Killer Cells Is Associated With Control of Hepatitis C Virus Infection. *Gastroenterology* **138,** 1885–1897.e10 (May 2010).

55. Peltier, D. C., Simms, A., Farmer, J. R. & Miller, D. J. Human Neuronal Cells Possess Functional Cytoplasmic and TLR-Mediated Innate Immune Pathways Influenced by Phosphatidylinositol-3 Kinase Signaling. *The Journal of Immunology* **184,** 7010–7021 (May 2010).

56. Greenwell-Wild, T. *et al.* Interleukin-27 inhibition of HIV-1 involves an intermediate induction of type I interferon. *Blood* **114,** 1864–1874 (June 2009).

57. Giovannini-Chami, L. *et al.* Distinct epithelial gene expression phenotypes in childhood respiratory allergy. *European Respiratory Journal* **39,** 1197–1205 (Oct. 2011).

58. Shapira, S. D. *et al.* A Physical and Regulatory Map of Host-Influenza Interactions Reveals Pathways in H1N1 Infection. *Cell* **139,** 1255–1267 (Dec. 2009).

59. Guo, Y. *et al.* Effects of Siltuximab on the IL-6-Induced Signaling Pathway in Ovarian Cancer. *Clinical Cancer Research* **16,** 5759–5769 (Aug. 2010).

60. Thomas, E. *et al.* HCV Infection Induces a Unique Hepatic Innate Immune Response Associated With Robust Production of Type III Interferons. *Gastroenterology* **142,** 978–988 (Apr. 2012).

61. Haglund, F. *et al.* Prolactin Receptor in Primary Hyperparathyroidism – Expression, Functionality and Clinical Correlations. *PLoS ONE* **7** (ed Mancini, M. A.) e36448 (May 2012).

62. Johnson-Huang, L. M. *et al.* A Single Intradermal Injection of IFN- Induces an Inflammatory State in Both Non-Lesional Psoriatic and Healthy Skin. *Journal of Investigative Dermatology* **132,** 1177–1187 (Apr. 2012).

63. Swindell, W. R. *et al.* Heterogeneity of Inflammatory and Cytokine Networks in Chronic Plaque Psoriasis. *PLoS ONE* **7** (ed Kontoyiannis, D. L.) e34594 (Mar. 2012).

64. Dill, M. T. *et al.* Interferon-–Stimulated Genes, but Not USP18, Are Expressed in Livers of Patients With Acute Hepatitis C. *Gastroenterology* **143,** 777–786.e6 (Sept. 2012).

65. Teles, R. M. B. *et al.* Type I Interferon Suppresses Type II Interferon-Triggered Human Anti-Mycobacterial Responses. *Science* **339,** 1448–1453 (Feb. 2013).

66. Sadras, T. *et al.* Interleukin-3-mediated regulation of -catenin in myeloid transformation and acute myeloid leukemia. *Journal of Leukocyte Biology* **96,** 83–91 (Mar. 2014).

67. Happold, C. *et al.* Interferon- Induces Loss of Spherogenicity and Overcomes Therapy Resistance of Glioblastoma Stem Cells. *Molecular Cancer Therapeutics* **13,** 948–961 (Feb. 2014).

68. Jin, S. H., Choi, D., Chun, Y.-J. & Noh, M. Keratinocyte-derived IL-24 plays a role in the positive feedback regulation of epidermal inflammation in response to environmental and endogenous toxic stressors. *Toxicology and Applied Pharmacology* **280,** 199–206 (Oct. 2014).

69. Abiko, K. *et al.* IFN- from lymphocytes induces PD-L1 expression and promotes progression of ovarian cancer. *British Journal of Cancer* **112,** 1501–1509 (Mar. 2015).

70. Jura, J. *et al.* Identification of interleukin-1 and interleukin-6-responsive genes in human monocyte-derived macrophages using microarrays. *Biochimica et Biophysica Acta (BBA) - Gene Regulatory Mechanisms* **1779,** 383–389 (June 2008).

71. Marzec, M. *et al.* Differential Effects of Interleukin-2 and Interleukin-15 versus Interleukin-21 on CD4 Cutaneous T-Cell Lymphoma Cells. *Cancer Research* **68,** 1083–1091 (Feb. 2008).

72. Moserle, L. *et al.* The Side Population of Ovarian Cancer Cells Is a Primary Target of IFN- Antitumor Effects. *Cancer Research* **68,** 5658–5668 (July 2008).

73. Broggini, M. RAS/RAF/MEK Inhibitors in Oncology. *Current Medicinal Chemistry* **19,** 1164–1176 (Mar. 2012).

74. English, J. M. & Cobb, M. H. Pharmacological inhibitors of MAPK pathways. *Trends in Pharmacological Sciences* **23,** 40–45 (Jan. 2002).

75. Akinleye, A., Furqan, M., Mukhi, N., Ravella, P. & Liu, D. MEK and the inhibitors: from bench to bedside. *Journal of Hematology & Oncology* **6,** 27 (2013).

76. Tsai, J. *et al.* Discovery of a selective inhibitor of oncogenic B-Raf kinase with potent antimelanoma activity. *Proceedings of the National Academy of Sciences* **105,** 3041–3046 (Feb. 2008).

77. Pratilas, C. A. *et al.* V600EBRAF is associated with disabled feedback inhibition of RAF-MEK signaling and elevated transcriptional output of the pathway. *Proceedings of the National Academy of Sciences* **106,** 4519–4524 (Feb. 2009).

78. Hoeflich, K. P. *et al.* In vivo Antitumor Activity of MEK and Phosphatidylinositol 3-Kinase Inhibitors in Basal-Like Breast Cancer Models. *Clinical Cancer Research* **15,** 4649–4664 (June 2009).

79. Stinson, S. *et al.* TRPS1 Targeting by miR-221/222 Promotes the Epithelial-to-Mesenchymal Transition in Breast Cancer. *Science Signaling* **4,** ra41–ra41 (June 2011).

80. Hoeflich, K. P. *et al.* Antitumor Efficacy of the Novel RAF Inhibitor GDC-0879 Is Predicted by BRAFV600E Mutational Status and Sustained Extracellular Signal-Regulated Kinase/Mitogen-Activated Protein Kinase Pathway Suppression. *Cancer Research* **69,** 3042–3051 (Mar. 2009).

81. Chang, J. T. *et al.* A Genomic Strategy to Elucidate Modules of Oncogenic Pathway Signaling Networks. *Molecular Cell* **34,** 104–114 (Apr. 2009).

82. Dumesic, P. A., Scholl, F. A., Barragan, D. I. & Khavari, P. A. Erk1/2 MAP kinases are required for epidermal G2/M progression. *The Journal of Cell Biology* **185,** 409–422 (May 2009).

83. Packer, L. M., East, P., Reis-Filho, J. S. & Marais, R. Identification of direct transcriptional targets ofV600EBRAF/MEK signalling in melanoma. *Pigment Cell & Melanoma Research* **22,** 785–798 (Dec. 2009).

84. Joseph, E. W. *et al.* The RAF inhibitor PLX4032 inhibits ERK signaling and tumor cell proliferation in a V600E BRAF-selective manner. *Proceedings of the National Academy of Sciences* **107,** 14903–14908 (July 2010).

85. Nazarian, R. *et al.* Melanomas acquire resistance to B-RAF(V600E) inhibition by RTK or N-RAS upregulation. *Nature* **468,** 973–977 (Nov. 2010).

86. Christian, S. L. *et al.* Suppression of IFN-Induced Transcription Underlies IFN Defects Generated by Activated Ras/MEK in Human Cancer Cells. *PLoS ONE* **7** (ed Subbiah, E.) e44267 (Sept. 2012).

87. Greger, J. G. *et al.* Combinations of BRAF, MEK, and PI3K/mTOR Inhibitors Overcome Acquired Resistance to the BRAF Inhibitor GSK2118436 Dabrafenib, Mediated by NRAS or MEK Mutations. *Molecular Cancer Therapeutics* **11,** 909–920 (Mar. 2012).

88. Balko, J. M. *et al.* Activation of MAPK Pathways due to DUSP4 Loss Promotes Cancer Stem Cell-like Phenotypes in Basal-like Breast Cancer. *Cancer Research* **73,** 6346–6358 (Aug. 2013).

89. Parmenter, T. J. *et al.* Response of BRAF-Mutant Melanoma to BRAF Inhibition Is Mediated by a Network of Transcriptional Regulators of Glycolysis. *Cancer Discovery* **4,** 423–433 (Jan. 2014).

90. Gysin, S., Paquette, J. & McMahon, M. Analysis of mRNA Profiles after MEK1/2 Inhibition in Human Pancreatic Cancer Cell Lines Reveals Pathways Involved in Drug Sensitivity. *Molecular Cancer Research* **10,** 1607–1619 (July 2012).

91. Gazel, A., Nijhawan, R. I., Walsh, R. & Blumenberg, M. Transcriptional profiling defines the roles of ERK and p38 kinases in epidermal keratinocytes. *Journal of Cellular Physiology* **215,** 292–308 (May 2008).

92. Webster, D. E. *et al.* Enhancer-targeted genome editing selectively blocks innate resistance to oncokinase inhibition. *Genome Research* **24,** 751–760 (Jan. 2014).

93. Fitzgerald, D. *et al.* Tumour necrosis factor- (TNF-) increases nuclear factor B (NFB) activity in and interleukin-8 (IL-8) release from bovine mammary epithelial cells. *Veterinary Immunology and Immunopathology* **116,** 59–68 (Mar. 2007).

94. Qin, H. LPS induces CD40 gene expression through the activation of NF- B and STAT-1 in macrophages and microglia. *Blood* **106,** 3114–3122 (Nov. 2005).

95. Saadane, A., Masters, S., DiDonato, J., Li, J. & Berger, M. Parthenolide Inhibits IB Kinase, NF-B Activation, and Inflammatory Response in Cystic Fibrosis Cells and Mice. *American Journal of Respiratory Cell and Molecular Biology* **36,** 728–736 (June 2007).

96. Ikeda, Y., Adachi, Y., Ishibashi, K.-i., Miura, N. & Ohno, N. Activation of Toll-Like Receptor-Mediated NF- by Zymosan-Derived Water-Soluble Fraction: Possible Contribution of Endotoxin-Like Substances. *Immunopharmacology and Immunotoxicology* **27,** 285–298 (Jan. 2005).

97. Garć, M. G. *et al.* Inhibition of NF-B activity by BAY 11-7082 increases apoptosis in multidrug resistant leukemic T-cell lines. *Leukemia Research* **29,** 1425–1434 (Dec. 2005).

98. Yu, W. *et al.* Cyclin T1-Dependent Genes in Activated CD4 T and Macrophage Cell Lines Appear Enriched in HIV-1 Co-Factors. *PLoS ONE* **3** (ed KewalRamani, V. N.) e3146 (Sept. 2008).

99. Ceppi, M. *et al.* Ribosomal protein mRNAs are translationally-regulated during human dendritic cells activation by LPS. *Immunome Research* **5,** 5 (2009).

100. Chhikara, M. *et al.* Carbon Monoxide Blocks Lipopolysaccharide-Induced Gene Expression by Interfering with Proximal TLR4 to NF-B Signal Transduction in Human Monocytes. *PLoS ONE* **4** (ed Aziz, R. K.) e8139 (Dec. 2009).

101. Leyva-Illades, D., Cherla, R. P., Galindo, C. L., Chopra, A. K. & Tesh, V. L. Global Transcriptional Response of Macrophage-Like THP-1 Cells to Shiga Toxin Type 1. *Infection and Immunity* **78,** 2454–2465 (Mar. 2010).

102. Schmelzer, C. & Döring, F. Identification of LPS-inducible genes downregulated by ubiquinone in human THP-1 monocytes. *BioFactors* **36,** 222–228 (Apr. 2010).

103. Dawson, K. *et al.* Modulation of blood cell gene expression by DHA supplementation in hypertriglyceridemic men. *The Journal of Nutritional Biochemistry* **23,** 616–621 (June 2012).

104. Kotz, K. T. *et al.* Clinical microfluidics for neutrophil genomics and proteomics. *Nature Medicine* **16,** 1042–1047 (Aug. 2010).

105. Jansen, B. J. *et al.* MicroRNA genes preferentially expressed in dendritic cells contain sites for conserved transcription factor binding motifs in their promoters. *BMC Genomics* **12.** doi:10.1186/1471-2164-12-330. https://doi.org/10.1186/1471-2164-12-330 (June 2011).

106. Yu, W.-H., Hu, H., Zhou, Q., Xia, Y. & Amar, S. Bioinformatics Analysis of Macrophages Exposed to Porphyromonas gingivalis: Implications in Acute vs. Chronic Infections. *PLoS ONE* **5** (ed Heimesaat, M. M.) e15613 (Dec. 2010).

107. Smith, S. M. *et al.* Tribbles 3: A Novel Regulator of TLR2-Mediated Signaling in Response to Helicobacter pylori Lipopolysaccharide. *The Journal of Immunology* **186,** 2462–2471 (Jan. 2011).

108. Maneck, M., Schrader, A., Kube, D. & Spang, R. Genomic data integration using guided clustering. *Bioinformatics* **27,** 2231–2238 (June 2011).

109. Iglesias, M. J. *et al.* Combined Chromatin and Expression Analysis Reveals Specific Regulatory Mechanisms within Cytokine Genes in the Macrophage Early Immune Response. *PLoS ONE* **7** (ed Spilianakis, C. B.) e32306 (Feb. 2012).

110. de Kleijn, S. *et al.* IFN--Stimulated Neutrophils Suppress Lymphocyte Proliferation through Expression of PD-L1. *PLoS ONE* **8** (ed Ryffel, B.) e72249 (Aug. 2013).

111. Schrader, A. *et al.* Global gene expression changes of in vitro stimulated human transformed germinal centre B cells as surrogate for oncogenic pathway activation in individual aggressive B cell lymphomas. *Cell Communication and Signaling* **10,** 43 (2012).

112. Lowe, J. M. *et al.* p53 and NF- B Coregulate Proinflammatory Gene Responses in Human Macrophages. *Cancer Research* **74,** 2182–2192 (Apr. 2014).

113. Guijarro-Muñoz, I., Compte, M., Álvarez-Cienfuegos, A., Álvarez-Vallina, L. & Sanz, L. Lipopolysaccharide Activates Toll-like Receptor 4 (TLR4)-mediated NF-B Signaling Pathway and Proinflammatory Response in Human Pericytes. *Journal of Biological Chemistry* **289,** 2457–2468 (Dec. 2013).

114. Allantaz-Frager, F. *et al.* Identification of Biomarkers of Response to IFNg during Endotoxin Tolerance: Application to Septic Shock. *PLoS ONE* **8** (ed Caldwell, C. C.) e68218 (July 2013).

115. McIntyre, B. A. *et al.* Innate immune response of human pluripotent stem cell-derived airway epithelium. *Innate Immunity* **21,** 504–511 (Sept. 2014).

116. Hassane, D. C. *et al.* Discovery of agents that eradicate leukemia stem cells using an in silico screen of public gene expression data. *Blood* **111,** 5654–5662 (Feb. 2008).

117. Nelson, W. G. & Kastan, M. B. DNA strand breaks: the DNA template alterations that trigger p53-dependent DNA damage response pathways. *Molecular and Cellular Biology* **14,** 1815–1823 (Mar. 1994).

118. Dulić, V. *et al.* p53-dependent inhibition of cyclin-dependent kinase activities in human fibroblasts during radiation-induced G1 arrest. *Cell* **76,** 1013–1023 (Mar. 1994).

119. Shen, H. & Maki, C. G. Pharmacologic Activation of p53 by Small-Molecule MDM2 Antagonists. *Current Pharmaceutical Design* **17,** 560–568 (Feb. 2011).

120. Vassilev, L. T. In Vivo Activation of the p53 Pathway by Small-Molecule Antagonists of MDM2. *Science* **303,** 844–848 (Feb. 2004).

121. Issaeva, N. *et al.* Small molecule RITA binds to p53, blocks p53–HDM-2 interaction and activates p53 function in tumors. *Nature Medicine* **10,** 1321–1328 (Nov. 2004).

122. Pabla, N., Huang, S., Mi, Q.-S., Daniel, R. & Dong, Z. ATR-Chk2 Signaling in p53 Activation and DNA Damage Response during Cisplatin-induced Apoptosis. *Journal of Biological Chemistry* **283,** 6572–6583 (Dec. 2007).

123. Martin, L. P., Hamilton, T. C. & Schilder, R. J. Platinum Resistance: The Role of DNA Repair Pathways. *Clinical Cancer Research* **14,** 1291–1295 (Mar. 2008).

124. Grinkevich, V. V. *et al.* Ablation of Key Oncogenic Pathways by RITA-Reactivated p53 Is Required for Efficient Apoptosis. *Cancer Cell* **15,** 441–453 (May 2009).

125. Iorio, F. *et al.* Discovery of drug mode of action and drug repositioning from transcriptional responses. *Proceedings of the National Academy of Sciences* **107,** 14621–14626 (Aug. 2010).

126. Goldstein, I. *et al.* Chemotherapeutic agents induce the expression and activity of their clearing enzyme CYP3A4 by activating p53. *Carcinogenesis* **34,** 190–198 (Oct. 2012).

127. Saligan, L. *et al.* Upregulation of -synuclein during localized radiation therapy signals the association of cancer-related fatigue with the activation of inflammatory and neuroprotective pathways. *Brain, Behavior, and Immunity* **27,** 63–70 (Jan. 2013).

128. Hussner, J. *et al.* Regulation of Interferon-Inducible Proteins by Doxorubicin via Interferon -Janus Tyrosine Kinase-Signal Transducer and Activator of Transcription Signaling in Tumor Cells. *Molecular Pharmacology* **81,** 679–688 (Feb. 2012).

129. Christgen, M. *et al.* IPH-926 lobular breast cancer cells harbor a p53 mutant with temperature-sensitive functional activity and allow for profiling of p53-responsive genes. *Laboratory Investigation* **92,** 1635–1647 (Sept. 2012).

130. Menendez, D. *et al.* Diverse stresses dramatically alter genome-wide p53 binding and transactivation landscape in human cancer cells. *Nucleic Acids Research* **41,** 7286–7301 (June 2013).

131. McDade, S. S. *et al.* Genome-wide characterization reveals complex interplay between TP53 and TP63 in response to genotoxic stress. *Nucleic Acids Research* **42,** 6270–6285 (May 2014).

132. Janku, F. *et al.* PIK3CA Mutation H1047R Is Associated with Response to PI3K/AKT/mTOR Signaling Pathway Inhibitors in Early-Phase Clinical Trials. *Cancer Research* **73,** 276–284 (Oct. 2012).

133. Vara, J. Á. F. *et al.* PI3K/Akt signalling pathway and cancer. *Cancer Treatment Reviews* **30,** 193–204 (Apr. 2004).

134. Rodon, J., Dienstmann, R., Serra, V. & Tabernero, J. Development of PI3K inhibitors: lessons learned from early clinical trials. *Nature Reviews Clinical Oncology* **10,** 143–153 (Feb. 2013).

135. Gharbi, S. *et al.* Exploring the specificity of the PI3K family inhibitor LY294002. *Biochemical Journal* **404,** 15–21 (May 2007).

136. Yuan, T. L. & Cantley, L. C. PI3K pathway alterations in cancer: variations on a theme. *Oncogene* **27,** 5497–5510 (Sept. 2008).

137. Brachmann, S. M. *et al.* Characterization of the Mechanism of Action of the Pan Class I PI3K Inhibitor NVP-BKM120 across a Broad Range of Concentrations. *Molecular Cancer Therapeutics* **11,** 1747–1757 (May 2012).

138. Wojtalla, A. *et al.* Targeting the Phosphoinositide 3-Kinase p110- Isoform Impairs Cell Proliferation, Survival, and Tumor Growth in Small Cell Lung Cancer. *Clinical Cancer Research* **19,** 96–105 (Nov. 2012).

139. Santo, E. E. *et al.* FOXO3a Is a Major Target of Inactivation by PI3K/AKT Signaling in Aggressive Neuroblastoma. *Cancer Research* **73,** 2189–2198 (Feb. 2013).

140. Kennedy, A. *et al.* Activation of the PIK3CA/AKT Pathway Suppresses Senescence Induced by an Activated RAS Oncogene to Promote Tumorigenesis. *Molecular Cell* **42,** 36–49 (Apr. 2011).

141. Rosich, L. *et al.* Dual PI3K/mTOR inhibition is required to effectively impair microenvironment survival signals in mantle cell lymphoma. *Oncotarget* **5,** 6788–6800 (July 2014).

142. Kim, J.-S., Lee, C., Bonifant, C. L., Ressom, H. & Waldman, T. Activation of p53-Dependent Growth Suppression in Human Cells by Mutations in PTEN or PIK3CA. *Molecular and Cellular Biology* **27,** 662–677 (Oct. 2006).

143. Chan, G., Bivins-Smith, E. R., Smith, M. S. & Yurochko, A. D. Transcriptome Analysis of NF- B- and Phosphatidylinositol 3-Kinase-Regulated Genes in Human Cytomegalovirus-Infected Monocytes. *Journal of Virology* **82,** 1040–1046 (Nov. 2007).

144. Annes, J. P. Making sense of latent TGFbeta activation. *Journal of Cell Science* **116,** 217–224 (Jan. 2003).

145. ten Dijke, P. & Hill, C. S. New insights into TGF-–Smad signalling. *Trends in Biochemical Sciences* **29,** 265–273 (May 2004).

146. Guo, X. & Wang, X.-F. Signaling cross-talk between TGF-/BMP and other pathways. *Cell Research* **19,** 71–88 (Jan. 2009).

147. Grundberg, E. *et al.* Systematic assessment of the human osteoblast transcriptome in resting and induced primary cells. *Physiological Genomics* **33,** 301–311 (Mar. 2008).

148. Nili, M., Shinde, U. & Rotwein, P. Soluble Repulsive Guidance Molecule c/Hemojuvelin Is a Broad Spectrum Bone Morphogenetic Protein (BMP) Antagonist and Inhibits both BMP2- and BMP6-mediated Signaling and Gene Expression. *Journal of Biological Chemistry* **285,** 24783–24792 (June 2010).

149. Gunaratne, A., Chan, E., El-Chabib, T. H., Carter, D. & Guglielmo, G. M. D. aPKC alters the TGF response in NSCLC cells through both Smad-dependent and Smad-independent pathways. *Journal of Cell Science* **128,** 487–498 (Dec. 2014).

150. Trompouki, E. *et al.* Lineage Regulators Direct BMP and Wnt Pathways to Cell-Specific Programs during Differentiation and Regeneration. *Cell* **147,** 577–589 (Oct. 2011).

151. Kennedy, B. A. *et al.* ChIP-seq Defined Genome-Wide Map of TGF/SMAD4 Targets: Implications with Clinical Outcome of Ovarian Cancer. *PLoS ONE* **6** (ed Tan, P.) e22606 (July 2011).

152. Luo, W., Friedman, M. S., Hankenson, K. D. & Woolf, P. J. Time series gene expression profiling and temporal regulatory pathway analysis of BMP6 induced osteoblast differentiation and mineralization. *BMC Systems Biology* **5,** 82 (2011).

153. Yeung, T.-L. *et al.* TGF- Modulates Ovarian Cancer Invasion by Upregulating CAF-Derived Versican in the Tumor Microenvironment. *Cancer Research* **73,** 5016–5028 (July 2013).

154. Walenda, G. *et al.* TGF-beta1 Does Not Induce Senescence of Multipotent Mesenchymal Stromal Cells and Has Similar Effects in Early and Late Passages. *PLoS ONE* **8** (ed Pesce, M.) e77656 (Oct. 2013).

155. Peng, J. *et al.* The BMP signaling pathway leads to enhanced proliferation in serous ovarian cancer-A potential therapeutic target. *Molecular Carcinogenesis* **55,** 335–345 (Feb. 2015).

156. Faiz, A. *et al.* Latrophilin receptors: novel bronchodilator targets in asthma. *Thorax* **72,** 74–82 (June 2016).

157. Chen, G. TNF-R1 Signaling: A Beautiful Pathway. *Science* **296,** 1634–1635 (May 2002).

158. Aggarwal, B. B. Signalling pathways of the TNF superfamily: a double-edged sword. *Nature Reviews Immunology* **3,** 745–756 (Sept. 2003).

159. Frasor, J. *et al.* Positive Cross-Talk between Estrogen Receptor and NF- B in Breast Cancer. *Cancer Research* **69,** 8918–8925 (Nov. 2009).

160. Pandey, A. K., Munjal, N. & Datta, M. Gene Expression Profiling and Network Analysis Reveals Lipid and Steroid Metabolism to Be the Most Favored by TNF in HepG2 Cells. *PLoS ONE* **5** (ed Cao, Y.) e9063 (Feb. 2010).

161. Takahashi, E. *et al.* Tumor Necrosis Factor- Regulates Transforming Growth Factor--dependent Epithelial-Mesenchymal Transition by Promoting Hyaluronan-CD44-Moesin Interaction. *Journal of Biological Chemistry* **285,** 4060–4073 (Dec. 2009).

162. Wollbold, J. *et al.* Adapted Boolean network models for extracellular matrix formation. *BMC Systems Biology* **3,** 77 (2009).

163. Lo, K. A. *et al.* Genome-Wide Profiling of H3K56 Acetylation and Transcription Factor Binding Sites in Human Adipocytes. *PLoS ONE* **6** (ed Tjwa, M.) e19778 (June 2011).

164. Viemann, D. *et al.* TNF induces distinct gene expression programs in microvascular and macrovascular human endothelial cells. *Journal of Leukocyte Biology* **80,** 174–185 (May 2006).

165. Jin, F., Li, Y., Ren, B. & Natarajan, R. PU.1 and C/EBP synergistically program distinct response to NF- B activation through establishing monocyte specific enhancers. *Proceedings of the National Academy of Sciences* **108,** 5290–5295 (Mar. 2011).

166. Inglés-Esteve, J. *et al.* Inhibition of Specific NF-B Activity Contributes to the Tumor Suppressor Function of 14-3-3 in Breast Cancer. *PLoS ONE* **7** (ed Lo, K.-W.) e38347 (May 2012).

167. Smiljanovic, B. *et al.* The multifaceted balance of TNF- and type I/II interferon responses in SLE and RA: how monocytes manage the impact of cytokines. *Journal of Molecular Medicine* **90,** 1295–1309 (May 2012).

168. Wang, C. Q. *et al.* IL-17 and TNF Synergistically Modulate Cytokine Expression while Suppressing Melanogenesis: Potential Relevance to Psoriasis. *Journal of Investigative Dermatology* **133,** 2741–2752 (Dec. 2013).

169. Saben, J. *et al.* Early growth response protein-1 mediates lipotoxicity-associated placental inflammation: role in maternal obesity. *AJP: Endocrinology and Metabolism* **305,** E1–E14 (Apr. 2013).

170. Cooks, T. *et al.* Mutant p53 Prolongs NF-B Activation and Promotes Chronic Inflammation and Inflammation-Associated Colorectal Cancer. *Cancer Cell* **23,** 634–646 (May 2013).

171. Martinez-Cingolani, C. *et al.* Human blood BDCA-1 dendritic cells differentiate into Langerhans-like cells with thymic stromal lymphopoietin and TGF-. *Blood* **124,** 2411–2420 (Aug. 2014).

172. Cookson, V. J. *et al.* Binding loci of RelA-containing nuclear factor-kappaB dimers in promoter regions of PHM1-31 myometrial smooth muscle cells. *Molecular Human Reproduction* **21,** 865–883 (Sept. 2015).

173. Barker, K. *et al.* Transcriptome Profile of the Vascular Endothelial Cell Response toCandida albicans. *The Journal of Infectious Diseases* **198,** 193–202 (July 2008).

174. Lavrik, I. Death receptor signaling. *Journal of Cell Science* **118,** 265–267 (Jan. 2005).

175. Lessene, G., Czabotar, P. E. & Colman, P. M. BCL-2 family antagonists for cancer therapy. *Nature Reviews Drug Discovery* **7,** 989–1000 (Dec. 2008).

176. Souers, A. J. *et al.* ABT-199, a potent and selective BCL-2 inhibitor, achieves antitumor activity while sparing platelets. *Nature Medicine* **19,** 202–208 (Jan. 2013).

177. Olesen, M., Skov, V., Mechta, M., Mumm, B. H. & Rasmussen, L. M. No influence of OPG and its ligands, RANKL and TRAIL, on proliferation and regulation of the calcification process in primary human vascular smooth muscle cells. *Molecular and Cellular Endocrinology* **362,** 149–156 (Oct. 2012).

178. Urtishak, K. A. *et al.* Potent obatoclax cytotoxicity and activation of triple death mode killing across infant acute lymphoblastic leukemia. *Blood* **121,** 2689–2703 (Feb. 2013).

179. Opel, D. *et al.* Targeting inhibitor of apoptosis proteins by Smac mimetic elicits cell death in poor prognostic subgroups of chronic lymphocytic leukemia. *International Journal of Cancer* **137,** 2959–2970 (July 2015).

180. McMahon, G. VEGF Receptor Signaling in Tumor Angiogenesis. *The Oncologist* **5,** 3–10 (Apr. 2000).

181. Fredriksson, L., Li, H. & Eriksson, U. The PDGF family: four gene products form five dimeric isoforms. *Cytokine & Growth Factor Reviews* **15,** 197–204 (Aug. 2004).

182. Rifkin, D. B. Recent developments in the cell biology of basic fibroblast growth factor. *The Journal of Cell Biology* **109,** 1–6 (July 1989).

183. Cross, M. J. & Claesson-Welsh, L. FGF and VEGF function in angiogenesis: signalling pathways, biological responses and therapeutic inhibition. *Trends in Pharmacological Sciences* **22,** 201–207 (Apr. 2001).

184. Bukowski, R. M., Yasothan, U. & Kirkpatrick, P. Pazopanib. *Nature Reviews Drug Discovery* **9,** 17–18 (Jan. 2010).

185. Atkins, M. B., Yasothan, U. & Kirkpatrick, P. Everolimus. *Nature Reviews Drug Discovery* **8,** 535–536 (July 2009).

186. Saha, S., Islam, M., Shilpi, J. A. & Hasan, S. Inhibition of VEGF: a novel mechanism to control angiogenesis by Withania somnifera's key metabolite Withaferin A. *In Silico Pharmacology* **1,** 11 (2013).

187. Suehiro, J.-i., Hamakubo, T., Kodama, T., Aird, W. C. & Minami, T. Vascular endothelial growth factor activation of endothelial cells is mediated by early growth response-3. *Blood* **115,** 2520–2532 (Nov. 2009).

188. Shen, C. J. *et al.* Decreased cell adhesion promotes angiogenesis in a Pyk2-dependent manner. *Experimental Cell Research* **317,** 1860–1871 (Aug. 2011).

189. Ma, D. *et al.* Autocrine Platelet-Derived Growth Factor–Dependent Gene Expression in Glioblastoma Cells Is Mediated Largely by Activation of the Transcription Factor Sterol Regulatory Element Binding Protein and Is Associated with Altered Genotype and Patient Survival in Human Brain Tumors. *Cancer Research* **65,** 5523–5534 (July 2005).

190. Sim, F. J. *et al.* CD140a identifies a population of highly myelinogenic, migration-competent and efficiently engrafting human oligodendrocyte progenitor cells. *Nature Biotechnology* **29,** 934–941 (Sept. 2011).

191. Minami, T. *et al.* The Calcineurin-NFAT-Angiopoietin-2 Signaling Axis in Lung Endothelium Is Critical for the Establishment of Lung Metastases. *Cell Reports* **4,** 709–723 (Aug. 2013).

192. Yang, W. *et al.* Integration of proteomic and transcriptomic profiles identifies a novel PDGF-MYC network in human smooth muscle cells. *Cell Communication and Signaling* **12.** doi:10.1186/s12964-014-0044-z. https://doi.org/10.1186/s12964-014-0044-z (Aug. 2014).

193. Piccaluga, P. P. *et al.* Platelet-derived growth factor alpha mediates the proliferation of peripheral T-cell lymphoma cells via an autocrine regulatory pathway. *Leukemia* **28,** 1687–1697 (Jan. 2014).

194. Kupfer, P. *et al.* Novel application of multi-stimuli network inference to synovial fibroblasts of rheumatoid arthritis patients. *BMC Medical Genomics* **7.** doi:10.1186/1755-8794-7-40. https://doi.org/10.1186/1755-8794-7-40 (July 2014).

195. Antipova, A. A., Stockwell, B. R. & Golub, T. R. Gene expression-based screening for inhibitors of PDGFR signaling. *Genome Biology* **9,** R47 (2008).
