# Supplementary material for: Perturbation-response genes reveal signaling footprints in cancer gene expression
Source: Nat Commun. 2018 Jan 2;9:20. doi: 10.1038/s41467-017-02391-6 (PMC5750219; doi:10.1038/s41467-017-02391-6)
Supplement: Supplementary file 3 — Description of Additional Supplementary Files [file 41467_2017_2391_MOESM3_ESM.pdf]

**Description of Additional Supplementary Files**

File Name: Supplementary Data 1
Description: PROGENy model coefficients. The model matrix consists of 11 pathways and 1059 genes. Its coefficients are non-zero if the gene-pathway pair corresponds to the top 100 genes that were up-regulated upon stimulation of the pathway in a wide range of experiments. The value corresponds to the fitted z-score across experiments in our model. Only rows with at least one non-zero coefficient were included, as the rest is not used to infer pathway activity.

File Name: Supplementary Data 2
Description: Phophoprotein measurements. Table of background-corrected and ß-Tubulin normalized phosphoprotein intensity. Antibodies used for the following peptides: AKTS473, c-JunS63, ERK1/2T202, Y204/T185, Y187, IkBaS32,S36, JNKT183,Y185, MEK1S217,S221 and mTORS2448. Columns include replicate number, kind of perturbation, perturbation time, and readout for antibodies.
